# MCTR3 reprograms arthritic monocytes to upregulate Arginase-1 and exert pro-resolving and tissue-protective functions in experimental arthritis

**DOI:** 10.1016/j.ebiom.2022.103974

**Published:** 2022-04-14

**Authors:** Kimberly Pistorius, Lucy Ly, Patricia R. Souza, Esteban A. Gomez, Duco S. Koenis, Ana R. Rodriguez, Julie Foster, Jane Sosabowski, Mark Hopkinson, Vinothini Rajeeve, Bernd W. Spur, Andrew Pitsillides, Costantino Pitzalis, Jesmond Dalli

**Affiliations:** a William Harvey Research Institute, Barts and The London School of Medicine and Dentistry, Queen Mary University of London, Charterhouse Square, London, EC1M 6BQ UK; b Rowan University School of Osteopathic Medicine, Department of Cell Biology & Neuroscience, 2 Medical Centre Drive, Stratford NJ 08084, USA; c Centre for Cancer Biomarkers and Biotherapeutics, Barts Cancer Institute, Barts and The London School of Medicine and Dentistry, Queen Mary University of London, Charterhouse Square, London, EC1M 6BQ UK; d Department of Comparative Biomedical Sciences, Royal Veterinary College, London, UK; e Mass spectrometry Laboratory, Barts Cancer Institute, Queen Mary University of London, Charterhouse Square, London, EC1M 6BQ, United Kingdom; f Centre for Inflammation and Therapeutic Innovation, Queen Mary University of London, London, UK

**Keywords:** SPM, Resolution, Rheumatoid arthritis, Macrophages, Tissue repair

## Abstract

**Background:**

Rheumatoid arthritis (RA) is a progressive degenerative disorder that leads to joint destruction. Available treatments only target the inflammatory component with minimal impact on joint repair. We recently uncovered a previously unappreciated family of pro-resolving mediators, the maresin conjugate in tissue regeneration (MCTR), that display both immunoregulatory and tissue-protective activities. Thus, we queried whether the production of these autacoids is disrupted in RA patients and whether they can be useful in treating joint inflammation and promoting joint repair.

**Methods:**

Using a highly phenotyped RA cohort we evaluated plasma MCTR concentrations and correlated these to clinical markers of disease activity. To evaluate the immunoregulatory and tissue reparative activities we employed both *in vivo* models of arthritis and organ culture models.

**Findings:**

Herein, we observed that plasma MCTR3 concentrations were negatively correlated with joint disease activity and severity in RA patients. Evaluation of the mechanisms engaged by this mediator in arthritic mice demonstrated that MCTR3 reprograms monocytes to confer enduring joint protective properties. Single cell transcriptomic profiling and flow cytometric evaluation of macrophages from mice treated with MCTR3-reprogrammed monocytes revealed a role for Arginase-1 (Arg-1) in mediating their joint reparative and pro-resolving activities. Arg-1 inhibition reversed both the anti-arthritic and tissue reparative actions of MCTR3-reprogrammed monocytes.

**Interpretation:**

Our findings demonstrate that circulating MCTR3 levels are negatively correlated with disease in RA. When administered to mice *in vivo*, MCTR3 displayed both anti-inflammatory and joint reparative activities, protecting both cartilage and bone in murine arthritis. These activities were, at least in part, mediated *via* the reprogramming of mononuclear phagocyte responses.

**Funding:**

This work was supported by funding from the European Research Council (ERC) under the European Union's Horizon 2020 research and innovation programme (grant no: 677542) and the Barts Charity (grant no: MGU0343) to J.D. J.D. is also supported by a Sir Henry Dale Fellowship jointly funded by the Wellcome Trust and the Royal Society (grant 107613/Z/15/Z).

**Research in context**

**Evidence before this study**

Current therapeutic strategies used to treat patients with chronic inflammatory disorders are primarily targeted to curtail inflammation with limited abilities to engage reparative mechanisms. This is a major shortcoming of these treatments given that the inflammatory process in many chronic inflammatory disorders results in significant tissue and organ damage. One such example are patients with Rheumatoid Arthritis (RA), where the chronic inflammatory process leads to the destruction of joints leading to permanent disability.

**Added value of this study**

In the present studies we found that the circulating concentrations of the tissue protective factor Maresin Conjugate in Tissue Regeneration 3 (MCTR3) are negatively correlated with joint damage and disease in patients with RA. Evaluation of the activities of this mediator in models of joint inflammation demonstrated MCTR3 potently decreased joint inflammation and promoted tissue repair. These remarkable anti-inflammatory and tissue reparative activities of MCTR3 were, at least in part, mediated *via* the reprogramming of circulating monocytes to yield macrophages with enhanced anti-inflammatory and tissue reparative properties.

**Implications of all the available evidence**

Thus, these finding indicate that MCTR3 or molecules based on this mediator may provide previously unappreciated leads for the development of therapeutics that can target both the inflammatory component and promote tissue repair in chronic inflammatory conditions.

Alt-text: Unlabelled box

## Introduction

Rheumatoid arthritis (RA) is a chronic inflammatory disorder characterized by dysregulated immune activation and unremitting inflammation.^1^, ^2^, ^3^, ^4^, ^5^ This persistent inflammatory response is linked with a progressive destruction of joints, leading to substantial morbidity and a reduction in quality of life. Significant progress has been made in the last few decades in both the early diagnosis and treatment of patients with RA.^1^, ^2^, ^3^, ^4^, ^5^ In particular the development of biological drugs that target molecules linked with the propagation of arthritic inflammation, including tumour necrosis factor (TNF) and interleukin (IL)-6, has transformed the management of patients with this otherwise debilitating condition. Despite these major leaps forward in the treatment of patients with RA, available therapeutics only target the inflammatory component of the disease without rectifying the extensive damage that occurs within the joints.^6^ Furthermore, a significant portion of patients build resistance to many of these biological drugs with time, limiting their effectiveness. Thus, there is a need for the development of therapeutics that not only limit arthritic inflammation but also promote joint repair.

The immune system plays a central role in both the propagation of joint inflammation as well as the observed tissue destruction.^7^, ^8^, ^9^, ^10^, ^11^ Among the immune cells known to participate in both the onset and termination of rheumatoid arthritis are monocytes^10^, ^11^, ^12^ Findings made in experimental systems suggest that non-classical monocytes contribute to the onset of inflammatory arthritis, whereas classical monocytes are linked with the resolution of joint disease.^10^^,^^12^ Elegant investigations with human synovial tissues also suggest a role for monocyte-derived macrophages (MDM) in both the propagation and termination of human disease. Single cell RNA-sequencing (scRNA-seq) analysis of synovial cells from patients with active RA identified a subset of macrophages that displays a pro-inflammatory phenotype and promoted fibroblast invasiveness.^13^ On the other hand, scRNA-seq analysis of synovial macrophages from RA patients in remission identified two macrophage subsets that were enriched in genes linked with the negative regulation of inflammation and produce higher amounts of the specialized pro-resolving mediator (SPM) Resolvin D1.^14^ These observations are in line with recent findings detailing a link between disruptions in SPM pathways, including SPM formation and activity, and the onset and progression of inflammatory arthritis.^15^, ^16^, ^17^, ^18^

SPM are produced *via* the enzymatic conversion of essential fatty acids, primarily the omega-3 fatty acids eicosapentaenoic acid, n-3 docosapentaenoic acid and docosahexaenoic acid.^19^^,^^20^ These autacoids are classified into four main families termed as lipoxins, resolvins, protectins and maresins. Studies investigating endogenous mechanisms activated during self-limited inflammation that expedite the resolution of inflammation and promote the repair of damaged tissues uncovered previously unappreciated peptide-lipid conjugated mediators.^21^^,^^22^ This family of mediators, named as maresin conjugates in tissue regeneration (MCTR), is produced *via* the stereoselective conversion of the omega-3 fatty acid docosahexaenoic acid.^21^^,^^23^ MCTRs share key defining biological activities with other SPM, such as their ability to counter-regulate the production of inflammatory mediators, including eicosanoids and cytokines.^21^^,^^22^ They also limit activity of inflammatory molecules, where for example, in the lung they reduce LTD_4_-induced airway contraction and methacholine-induced hyperreactivity.^24^ MCTRs also exert potent tissue regenerative activities promoting tissue regeneration in surgically injured planaria.^21^^,^^22^

Given that MCTRs exert both inflammation-modulating and tissue-reparative activities in the present studies we evaluated whether MCTR levels are linked with disease severity in patients with RA, finding a significant negative correlation between plasma MCTR3 and joint disease activity. Administration of MCTR3 during peak of arthritic disease led to significant reduction in joint inflammation, as well as cartilage and bone protection. *Ex vivo* treatment of monocytes from arthritic mice with MCTR3 was sufficient to reprogram these cells to recapitulate the joint protective activities observed following the systemic administration of MCTR3. These results suggest that mononuclear phagocytes play a key role in the observed anti-arthritic and reparative activities exerted by this autacoid.

## Methods

### RA patient samples

Plasma samples were taken from RA patients who were DMARDs and steroid-naive, had symptoms duration <12 months, and fulfilled the ACR/EULAR 2010 classification criteria for RA and recruited into the Pathobiology of Early Arthritis Cohort (PEAC http://www.peac-mrc.mds.qmul.ac.uk). The PEAC cohort study was approved by the King's College Hospital Research Ethics Committee (REC 05/Q0703/198). Patients provided informed consent. Peripheral blood samples were obtained from patients recruited at Barts Health NHS Trust undergoing ultrasound (US)-guided synovial biopsy.^25^

### Targeted lipid mediator profiling

All samples were extracted using solid-phase extraction columns as in.^26^ In brief, samples were placed in ice-cold methanol containing deuterated internal standards (d_8_-5S-HETE, d_4_-LTB_4_, d_5_-LXA_4_, d_4_-PGE_2_, d_5_-RvD2, d_5_-MaR1, d_5_-MaR2, d_5_-RvD3, d_4_-RvE1, d_5_-17R-RvD1, d_5_-LTC_4_, d_5_-LTD_4_ and d_5_-LTE_4_) representing each chromatographic region of identified LMs. Following protein precipitation (-20°C for a minimum of 45 min), samples were centrifuged and supernatants extracted using an ExtraHera System (Biotage) using solid-phase extraction with Isolute C18 500 mg columns (Biotage). Methyl formate and methanol fractions were collected, brought to dryness and resuspended in phase (methanol/water, 1:1, vol/vol) for injection on a Shimadzu LC-20AD HPLC and a Shimadzu SIL-20AC autoinjector, paired with a QTrap 5500 or QTrap 6500+ (Sciex). In the analysis of mediators eluted in the methyl formate fraction, an Agilent Poroshell 120 EC-C18 column (100 mm × 4.6 mm × 2.7 μm) was kept at 50°C and mediators eluted using a mobile phase consisting of methanol/water/acetic acid of 20:80:0.01 (vol/vol/vol) that was ramped to 50:50:0.01 (vol/vol/vol) over 0.5 min and then to 80:20:0.01 (vol/vol/vol) from 2 min to 11 min, maintained till 14.5 min and then rapidly ramped to 98:2:0.01 (vol/vol/vol) for the next 0.1 min. This was subsequently maintained at 98:2:0.01 (vol/vol/vol) for 5.4 min, and the flow rate was maintained at 0.5 ml/min. QTrap 5500 was operated in negative ionization mode using a multiple reaction monitoring method. In the analysis of mediators eluted in the methanol fraction, an Agilent Poroshell 120 EC-C18 column (100 mm × 4.6 mm × 2.7 μm) was kept at 50°C and mediators eluted using a mobile phase consisting of methanol/water/acetic acid 55:45:0.5 (vol/vol/vol) over 5 min, that was ramped to 80:20:0.5 (vol/vol/vol) for 2 min, maintained at 80:20:0.5 (vol/vol/vol) for the successive 3 min and ramped to 98:2:0.5 (vol/vol/vol) over 3 min. This condition was kept for 3 min. QTrap 6500+ was operated in positive ionization mode using a multiple reaction monitoring method. Each lipid mediator was identified using established criteria, including:^1^ matching retention time to synthetic or authentic standards,^2^ ≥5 data points, and^3^ signal to noise ratio ≥5. Calibration curves were obtained for each mediator using synthetic compound mixtures at 0.78, 1.56, 3.12, 6.25, 12.5, 25, 50, 100, and 200 pg that gave linear calibration curves with an r^2^ values of 0.98–0.99.

### Putrescine quantification

To extract putrescine 50 μL of 200 ng/mL 1,4-diaminobutane-2,2,3,3-d_4_ dihydrochloride (d_4_-putrescine) and 250 μL 6% TCA were added to pulverized paws tissues, which were further homogenized using a Percellys 24 homogenizer. The resultant suspension was incubated on ice for 1 hour, then centrifuged at 10 000 x *g* for 10 minutes at 4°C. The supernatant was then collected and levels of putrescine and d_4_-putrescine were determined using a Shimadzu LC-20AD HPLC and a Shimadzu SIL-20AC autoinjector, paired with a QTrap 5500 (Sciex) operated in positive ionization mode using a multiple reaction monitoring method with the following transitions being used for the quantitation of putrescine – m/z 89>72 and d_4_-putrescine – m/z 97>76. An Agilent Poroshell 120 EC-C18 column (100 mm × 4.6 mm × 2.7 μm) kept at 30°C was used in the analysis of these molecules which were eluted using a mobile phase consisting of methanol/water/acetic acid 100:0:0.5 (vol/vol/vol) that was ramped to 0:100:0.5 (vol/vol/vol) over a 5 min period.

### Animal studies

10-week-old C57BL/6 mice (Charles River, UK), DBA/1 mice (Charles River, UK) and C57BL/6-Ly5.1 mice (Charles River, Italy) were used in the reported studies. All animal experiments were performed strictly in accordance with United Kingdom Home Office regulations (Guidance on the Operation of Animals, Scientific Procedures Act) and Laboratory Animal Science Association Guidelines (Guiding Principles on Good Practice for Animal Welfare and Ethical Review Bodies) and according to protocols detailed in a UK Home Office approved protocol (P998AB295). Mice were kept in specific pathogen free housing, food and water were provided *ad libitum* and kept with a 12h light-dark cycle, with lights on between 7:00 h and 19:00h. All animals were randomized to treatment of vehicle group prior.

### Inflammatory arthritis

*Glucose-6-phosphate isomerase (G6PI) peptide induced arthritis:* Antigen DBA/1 mice were immunised with a G6PI emulsion (100 µL/mouse), prepared by sonication of 10 µg G6PI peptide (Sequence: IWYINCFGCETHAML; Cambridge Peptides Ltd.) in 50 µL complete Freund's adjuvant (CFA) and 50 µL DPBS^−/−^ per mouse (Schubert et al., 2004), *via* intradermal injection at the base of the tail to initiate inflammatory arthritis. Arthritic DBA/1 mice were treated with 1 µg/mouse MCTR3 or vehicle (DPBS^−/−^ + 0.1 % EtOH) on day 24, 26 and 28 intravenously (i.v.). Paws were collected for microCT analysis and flow cytometry on day 36.

*K/BxN serum induced arthritis:* Arthritogenic K/BxN serum (100 µL/mouse) was administered *via* intraperitoneal (i.p.) injection to C57BL/6 mice on day 0 and 2 to induce self-resolving inflammatory arthritis.^27^ Disease severity was evaluated using a 26-point arthritic scoring system and ankle and pad oedema was measured daily using callipers.^16^ For femur head collection and BM cell isolations for *in vitro* cell cultures, mice were culled on Day 5. Otherwise, mice were administered a third K/BxN serum injection on either day 8 or 9 to prolong inflammatory arthritis. Mice were then treated *i.v*. with vehicle (DPBS^−/−^ + 0.1 % EtOH) or 1 µg/mouse MCTR3 on day 10, 12 and 14 and on day 25, paws were collected for histology and flow cytometry and blood was collected for ELISAs. MCTR3 was obtained *via* total organic synthesis as previously reported^28^ and its concentration was determined using its UV absorbance characteristics (see Figure S2 for compound validation).

In separate experiments, arthritis was initiated and prolonged as detailed above. On day 12, mice were treated *via i.v* injection with 2×10^6^ BM derived monocytes, obtained from arthritic C57BL/6 mice that were isolated and trained as detailed below. Paws were collected for flow cytometry, single cell RNA sequencing and histology on day 22.

In other experiments, arthritis was initiated and prolonged as above, on day 12 they were treated with vehicle or 200 µg N^ω^-Hydroxy-nor-L-arginine (nor-NOHA), an arginase 1 inhibitor, administered *via i.p*. injections daily. On Day 22 paws were collected for flow cytometry.

### Bone marrow isolations

Bone marrow cells were collected from naive DBA/1 mice, arthritic C57BL/6 mice on day 5 after the initial K/BxN injection or arthritic C57BL/6 mice on day 12 after the initiation of arthritis (see above). Briefly, femurs, tibiae and humeri were placed in 70 % EtOH and rinsed in DPBS^−/−^. The epiphysis was removed, and a 25G needle was used to flush the bone marrow with 2 mL DPBS^−/−^ per bone. Cells were dispersed gently with a 19G needle, filtered through a 70 µM strainer, centrifuged at 400 x *g* for 5 minutes at 4°C and suspended in DPBS^+/+^.

*For monocyte adoptive transfer experiments,* bone marrow-derived monocytes were isolated using EasySep™ Mouse Monocyte Isolation Kit (STEMCELL) according to manufacturer's instructions. Isolated monocytes from arthritic C57BL/6 mice were labelled with PKH67 Red Fluorescent Cell Linker kit (Sigma), following manufacturer's instructions. Monocytes were then incubated with either vehicle (DPBS^+/+^ + 0.01 % EtOH) or 1 nM MCTR3 for 90 min at 37°C. In separate experiments monocytes were first incubated with vehicle (DPBS^+/+^ + 0.1 % DMSO) or 10 µM RG108 (Sigma) for 15 min, prior to incubation with MCTR3 (1nM) or vehicle (DPBS^+/+^ + 0.01 % EtOH; 37°C).

In other experiments bone marrow cells were isolated from bone long bones collected from arthritic mice 5 days after the initiation of arthritis and seeded into 10 cm dishes. These were then incubated at 37°C for 45 minutes in DPBS^+/+^, the supernatant was removed and cells were washed with DPBS^−/−^ to remove non-adherent cells. Adherent cells were incubated with either 10 µM RG108 or a vehicle (DPBS^+/+^+ 0.1 % DMSO) for 45 minutes in 5 mL DMEM containing 1% penicillin and streptomycin (P/S), following which, 1 nM MCTR3 or vehicle (DPBS^+/+^ + 0.1 % EtOH) was added to the media. After 2 hours, an additional 5 mL DMEM containing 1 % P/S and 0.2 % FBS (for a final concentration of 0.1 % FBS) was added and the cells were incubated at 37°C at 5 % CO_2_ for a further 22 hours. Media was replaced with DMEM containing 1 % P/S, 10 % FBS and 20 ng/mL murine GM-CSF, and incubated for a further 4 days to allow for macrophage differentiation. Subsequently, macrophages were detached using 5 mM EDTA in DPBS^−/−^ and seeded, at 1.5×10^5^ cells/well, into 24-well Transwell plates in DMEM containing 1 % P/S and 10 % FBS for co-incubations with femoral heads. In separate experiments, BM derived monocytes were treated as described above, and following the replacement of media with DMEM containing 1 % P/S, 10 % FBS and 20 ng/mL murine GM-CSF, monocytes were allowed to differentiate for a further 6 days. Media was refreshed after 3 days.

To evaluate the role of Arg-1 in mediating the joint protective actions of monocyte derived macrophages, bone marrow monocytes were incubated with vehicle (DPBS + 0.1 % EtOH) or 1 nM MCTR3 in 5 mL DMEM containing 1% P/S for 2 hours at 37°C at 5 % CO_2_, after which an additional 5 mL DMEM containing 1 % P/S and 0.2 % FBS (for a final concentration of 0.1 % FBS) was added and the cells were incubated for a further 22 hours. Media was then replaced with DMEM containing 1 % P/S, 10 % FBS and 20 ng/mL murine GM-CSF and incubated for a further 2 days. Adherent cells were detached with 5 mM EDTA in DPBS^−/−^ and seeded into 24-well Transwell plates, at 2×10^5^ cells/well. Cells were then incubated in serum-free Accell siRNA delivery medium containing either 1 µM Accell anti-mouse *Arg1* siRNA SMARTpool or mouse control siRNA (Dharmacon) at 37°C at 5 % CO_2_ for 48 hours. Cells were washed with DPBS^−/−^ and DMEM containing 1 % P/S and 10 % FBS was added to the cells for co-incubations with femur heads.

### Femur head isolation and culture

Femur heads were collected as described in.^29^ Femur heads were removed from arthritic C57BL/6 mice, washed in 70 % EtOH, then in DPBS^−/−^, and incubated in 200 µL pre-warmed serum-free DMEM containing high glucose and 1% insulin-transferrin-selenium for 48 hours at 37°C and 5 % CO_2_. The medium was replaced with DMEM containing 10 % FBS and 10 ng/mL IL-1β and femur heads were incubated for a further 72 hours. These were then co-incubated with MDM that were prepared as detailed above for 48 hours at 37°C and 5 % CO_2_. Tissues were then collected and fixed in 10 % neutral buffered formalin (NBF) for histology.

### ELISAs

Mouse Cross Linked C-Telopeptide of Type-I Collagen (CTXI; Abbexa; 4 x dilution) was evaluated in plasma collected from arthritic C57BL/6 mice treated with vehicle or MCTR3, as per manufacturer's instruction.

### Histology

The femur heads and joints were fixed in 10 % NBF, for 72 hours and decalcified in 10 % EDTA (w/v) in DPBS^-^^/^^-^ for 2 weeks with shaking. The decalcified tissue was then processed and embedded in paraffin and 4-micron sections were cut.

### Safranin O

To assess cartilage deposition, tissues were incubated for 5 minutes with 0.1% Safranin O in 0.2 M acetic acid and 0.2 M sodium acetate, pH 4, washed in dH_2_O for 2 minutes and air-dried. For counter-staining, 0.05 % Light-green (GeneTex) in dH_2_O was added to the sections for 3 minutes and washed with dH_2_O twice for 2 minutes each. Sections were incubated twice in 100 % EtOH for 5 minutes each, briefly dipped in Histoclear, left to air dry and mounted with Entellan. Safranin O staining was imaged using either the EVOS microscope or Nanozoomer Slide scanner and NDP.view 2 software (Hamamatsu Photonics) and assessed with ImageJ 1.53 (Schneider *et al*., 2012).

### Collagen

Slides were heated at 50°C for 30 minutes, incubated in Histoclear twice, then twice in 100 % EtOH for 5 minutes each. Sections were washed in dH_2_O for 1 minute, air dried at room temperature, fixed in 4 % PFA for 5 minutes and washed in DPBS with on an orbital shaker twice for 5 minute intervals. For digestion of pepsin, slides were incubated in 0.02 % HCl for 7 minutes at 37°C, then for 20 minutes at 37°C in 3 mg/mL pepsin solution in 0.02 % HCl equilibrated to 37°C. This was washed twice in DPBS for 5 minutes on an orbital shaker, quenched by incubating in 50 nM ammonium chloride twice, each for 5 minutes on an orbital shaker. Sections were washed as above and blocked in 20 % FBS in DPBS for 1 hour at room temperature. Sections were incubated with primary mouse polyclonal anti-collagen type II (Merck Millipore; 1:500 in 20% FBS in DPBS) for 1 hour at room temperature in the dark, washed 3 times in DPBS for 10 minutes and incubated with secondary AF488 Goat anti-mouse IgG (1:400 in 20% FBS in DPBS) for 1 hour at room temperature. This was washed in PBS 3 times in the dark for 10 minutes each on an orbital shaker and incubated in the dark with efluoro570 anti-collagen X (1:200 in 20 % FBS in DPBS) at 4°C overnight. DPBS was used to wash the sections 3 times for 10 minutes each on an orbital shaker in the dark and slides were mounted with Mowiol with DAPI overnight.

### Micro-CT Analysis

The Siemens INVEON® PET/CT scanner (Siemens Preclinical Solutions, Knoxville, TN) with the Inveon Acquisition Workplace software was used perform micro-CT scans of the arthritic DBA mice knees at peak of disease on day 24 and following resolution of inflammatory arthritis, on day 35. All procedures were done in accordance with UK Home Office Regulations. Before scanning, the center offset and light/dark calibration was performed and a new workflow was created on the scanner. Mice were anesthetised with 3-5% inhalation anaesthesia, which was reduced to and maintained at 1.5% during scanning, at a rate of 1.5L/min . Mice were laid in prone position on a heating pad at 37°C to maintain body temperature during scanning. Scanning was performed at a voltage of 70kV, using an X-ray current of 500 μA and at an exposure time of 2000ms/projection for 360 projections. Hounsfield correction was used for image reconstruction.

To evaluate bone callus arthritic joints were collected 25 days after initiation of arthritis using K/BxN serum. Samples were wrapped in plastic film prior to scanning to prevent drying and scanned using a Skyscan 1172F (Bruker, Kontich, Belgium). The X-ray source was operated at 50kV and 200µA, using an Aluminium 0.5mm filter and an exposure time 960ms using a voxel size of 5µm. Projection images were reconstructed into tomograms using NRecon 1.7.3.1 (Bruker, Kontich, Belgium) and repositioned using Dataviewer 1.5.4 (Bruker, Kontich, Belgium) with bone analysis performed in CTAn 1.18.4 (Bruker, Kontich, Belgium). Volume rendered 3D visualisations were created using CTVox 3.3 (Bruker Kontich, Belgium).

### Leucocyte isolation from arthritic paws

Hind paw tissue digestion to isolate leukocytes from arthritic joints was performed as described in.^27^ Briefly, following the removal of skin and muscle, the hind paw was incubated in 15 mL digestion buffer (RPMI containing 0.5 μg/mL collagenase D and 40 μg/mL DNAse) at 37°C for 30 minutes with vigorous agitation. Liberated cells within the digestion buffer were passed through a 70 μM strainer into 10 mL 10 % FBS in RMPI on ice. The digestion incubation was repeated and the cell suspension volume was made up to 50 mL with 10 % FBS in RMPI. Cells were centrifuged at 400 *x g* for 10 minutes at 4°C and suspended in DPBS for flow cytometry.

### Gene expression

Tissue was homogenised using a a Percellys 24 homogenizer and an RNeasy Mini Kit (Qiagen) was used to extract RNA, as per manufacturers instruction. cDNA synthesis was achieved using Superscript III Reverse Transcriptase (Invitrogen), as per manufacturers instruction. QuantiTect Primer Assays (Qiagen) for mouse *KC, Il-6, Tnf-α, Mmp7, Fra-1, Dkk1, Lef1* and *sFrp-1* were used with SYBR green I fluorescent dye for real-time PCR (qRT-PCR) evaluation with the StepOne™ Real-Time PCR System (ThermoFisher). Target gene expression was expressed as a value relative to *Actb* expression.

### Flow cytometry

Isolated cells from arthritic paws were incubated with the following fluorescently conjugated antibodies: PE-Cy7 mouse anti-mouse CD64 (Biolegend), PerCP-Cy5.5 mouse anti-mouse CD64 (Biolegend), PE-Cy5 rat anti-mouse CD11b (Biolegend), PE-Texas Red rat anti-mouse CD11b (Biolegend), BV421 rat anti-mouse F4/80 (Biolegend), APC/Cy-7 rat anti-mouse F4/80 (Biolegend), BV711 rat anti-mouse MerTK (Biolegend) and/or PE-Cy7 Armenian hamster anti-mouse CD36 (Biolegend) at a dilution of 1:100 in DPBS^−/−^ with 0.02 % BSA for 30 minutes at 4°C. Cells were incubated with BD Fixation/Permeabilisation buffer solution and then with BD Permeabilisation Buffer, each for 20 minutes at room temperature. Intracellular staining was performed by incubating cells with BV 421 anti-mouse TGF-β1 (1:50 dilution, Biolegend), APC rat anti-mouse iNOS (1:100 dilution; Biolegend), PE sheep anti-mouse Arginase 1 (1:50 dilution, R&D) and polyclonal rabbit anti-DBL (1:100 dilution, Cell Signaling Technologies) for 30 minutes at 4°C. Rabbit anti-DBL was conjugated with PerCp/Cy5.5 using Abcam's PerCP/Cy5.5 Conjugation Kit, as per manufacturer's instructions. APC-Cy7 rat anti-mouse Ly6G (1:50 dilution, Biolegend) was also incubated with the cells following permeabilisation for intracellular staining. Cells were incubated with TruStain X to quench non-specific binding. Multiparameter analysis was performed with LSR Fortessa cell analyser (BD Biosciences) and analysed using FlowJo (Tree Star Inc., V10).

### Single-cell 3′ RNA sequencing

Following paw tissue digestion, as described above, live cells were obtained using EasySep^TM^ Dead Cell Removal (Annexin V) kit (STEM CELL) according to manufacturer's instruction. Cells were incubated with AF700 CD45 (Biolegend) at a dilution of 1:100 in DPBS^−/−^ with 0.02 % BSA for 30 minutes at 4°C. Non-specific staining was blocked with TruStain X. Cells were suspended in DPBS^−/−^ with 0.02 % BSA and the BD FACS Aria II was used sort for CD45 positive cells, which were collected in DPBS containing 0.1 % BSA for single cell sequencing.

### Sample quality control

8 single cell suspensions were prepared (one per mouse) and were assessed for cell number using the Luna FL automated cell counter (Logos biosystems, South Korea). Cells appeared intact and well distributed with an average count of 148 cells/µL.

### Single-cell library generation and RNA-sequencing

An equivalent volume of 4000 cells was loaded to the 10X Chromium™ Single Cell A Chip (PN-1000009) using the Chromium™ 3’ Library & Gel Bead Kit v2 (PN-120267) as described in the manufacturers user guide (10X Genomics, California, USA). GEMs were recovered from the chip and appeared opaque and uniform in colour. 14 cycles of cDNA amplification were performed on the purified GEM-RT product, and cDNA was examined for quality using the Agilent 2200 Tapestation with the High-sensitivity D5000 screentape and reagents (Agilent Technologies, Waldbronn, Germany), and the Qubit® 2.0 Fluorometer and Qubit dsDNA HS Assay Kit (Life Technologies, California, USA). 35 μL of cDNA were used to prepare the 10×3’RNA libraries and 12 cycles were used for sample index PCR. Final cleaned libraries were quantified using the Qubit® 2.0 Fluorometer and Qubit dsDNA HS Assay Kit and average fragment size checked using the Agilent D1000 screentape and reagents. The final pooled library was run on a NextSeq500 High-output v2.5 150-cycle kit with a 26[8]98 cycle configuration to generate 400 million read pairs in total.

### Preliminary data analysis

The 10X Genomics Cell Ranger pipeline was used to analyse the raw sequence data generated by the single cell RNA-seq (10x Genomics; https://support.10xgenomics.com/single-cell-gene-expression/software/). Shortly, the pipeline demultiplexes raw base call files generated by Illumina sequencers in FASTQ files and then aligns, filters, and counts (barcode and UMI) the reads. The alignment was done using STAR (https://github.com/alexdobin/STAR) and the *Mus musculus* genome (GRCm38)^30^ as the reference genome. The samples for each group were aggregated (to have a normalized set of cells per group) using “cell ranger aggr” function.

Using the Seurat R package^31^ quality control (QC) steps were performance to filter out low quality cells. Briefly, cells that contained fewer than 250 expressed genes (low-quality cells or empty droplets), more than 3000 expressed genes (cell doublets), more than 5% mitochondrial transcripts (indication of mitochondrial damage), and less than 500 UMI counts were removed. We considered genes as detectable if they were expresses in at least the number of cells that represent 20 % of smallest cell type population. After QC step, expression of each gene was normalized by total expression, then multiplied by a scale factor (1000) and log transformed. For dimensional reduction and clustering, highly variable genes were identified, and Principal Component Analysis (PCA) was performance to identify relevant principal components (PC) based on how much the standard deviation of the data was explained by them. PCs were used for data clustering (unsupervised graph-based clustering with resolution 0.5) and dimensionality reduction using the Uniform Manifold Approximation and Projection (UMAP) analysis. Data visualization was performance using the “DimPlot” function from Seurat R package.

### Differential gene expression analysis

Before differential gene expression analysis, distinct leukocyte subsets were identified based on the expression of specific markers (Table S1-3) and comparing the most expressed genes from each cluster with Single Cell Expression Atlas database (https://www.ebi.ac.uk/gxa/sc/home).

Differential gene expression analysis was performed using the likelihood ratio test from Edge R package (https://bioconductor.org/packages/release/bioc/html/edgeR.html). Statistical significance was considered with an adjusted (Benjamini-Hochberg procedure correction) *p-value* < 0.05.

### Pathway enrichment analysis

Pathway analysis was performed uploading the differentially expressed genes in NetworkAnalyst 3.0 (ORA enrichment visualization tab)^32^ and searching for the enriched pathways from KEGG^33^ and STRING^34^ (*p* value < 0.05, Fisher exact test followed by multiple comparison correction using Benjamini-Hochberg procedure) databases.

### Phosphoproteomic analysis

For evaluation of signalling pathways activated by MCTR3, monocytes were isolated from peripheral blood of healthy volunteers, incubated with GM-CSF (20ng/mL, in RPMI containing 10% human serum) for 7 days and then incubated with MCTR3 (1nM, in DPBS^+/+^).

Phosphoproteomics experiments were performed using mass spectrometry as reported.^35^^,^^36^ In brief, cells were lysed in 8M urea buffer and supplemented with phosphatase inhibitors (10 mM Na_3_VO_4_, 100 mM β-glycerol phosphate and 25 mM Na_2_H_2_P_2_O_7_ (Sigma)). Proteins were digested into peptides using trypsin as previously described.^37^^,^^38^ Phosphopeptides were enriched from total peptides by TiO2 chromatography essentially as reported previously^39^). Dried phosphopeptides were dissolved in 0.1% TFA and analysed by nanoflow ultimate 3000 RSL nano instrument which was coupled on-line to a Q Exactive plus mass spectrometer (Thermo Fisher Scientific). Gradient elution was from 3% to 35% buffer B in 120 min at a flow rate 300 nL/min with buffer A being used to balance the mobile phase (buffer A was 0.1% formic acid in water and B was 0.1% formic acid in acetonitrile). The spray voltage was 1.95 kV and the capillary temperature was set to 255 ºC. The Q-Exactive plus was operated in data dependent mode with one survey MS scan followed by 15 MS/MS scans. The full scans were acquired in the mass analyser at 375- 1500m/z with the resolution of 70 000, and the MS/MS scans were obtained with a resolution of 17 500.

MS raw files were converted into Mascot Generic Format using Mascot Distiller (version 2.5.1) and searched against the SwissProt database (release December 2015) restricted to human entries using the Mascot search daemon (version 2.5.0). Allowed mass windows were 10 ppm and 25 mmu for parent and fragment mass to charge values, respectively. Variable modifications included in searches were oxidation of methionine, pyro-glu (N-term) and phosphorylation of serine, threonine and tyrosine.

### Statistics

GraphPad Prism 8 (GraphPad Software, La Jolla, CA, USA) was used to assess differences between the groups. Spearman test was used to evaluate the correlation between the lipid mediators and disease parameters. To evaluate difference between experimental groups for group sizes less then 8 we used non-parametric tests. These include one-sample Wilcoxon signed rank test for normalized data between 2 groups, Mann-Whitney U test between 2 groups, a One-way ANOVA between 3 groups or Two-way ANOVA for time course analysis. No statistical analysis was performed to determine the sample size for *in vivo* experiments. Sample size was based on effect size observed in published studies evaluating the biological activities of other SPM^27^^,^^40^ and pilot results whereby the ability of MCTR3 to reduce both clinical scores and oedema was evaluated and found to be comparable to that in published studies. Therefore, we elected to employ similar sample sizes as those used in published studies. The experimenter was not blinded to treatment allocation. In select experiments mice that did not develop disease were excluded from further analysis.

### Role of funding source

The funders played no part in the design, data collection, data analyses, interpretation, writing of report or in the decision to publish the results.

## Results

### MCTR3 negatively correlates with joint disease in humans

Circulating lipid mediator concentrations are linked with peripheral organ disease activity, since these autacoids influence leukocyte recruitment and activation status.^41^, ^42^, ^43^, ^44^, ^45^ To establish whether there was a link between disease activity and MCTR concentrations in RA patients we investigated plasma levels of these molecules in relation to both systemic and joint disease activity markers. Plasma was obtained from The Pathobiology of Early Arthritis Cohort (PEAC), which is a highly phenotyped patient cohort of disease modifying anti-rheumatic drugs-naïve patients^25^ (See Table 1 for patient characteristics). Using lipid mediator profiling, we identified all three MCTRs in plasma from these patients. Notably, while concentrations of all three mediators were observed to display a negative correlation with joint disease activity (i.e. DAS28 scores), plasma C-reactive protein and erythrocyte sedimentation rate, only correlations between MCTR3 and these parameters were statically significant (Table 2). We also evaluated whether plasma concentrations of these mediators were linked with other clinical features of RA. Whilst plasma concentrations were not linked with joint disease pathotype or responsiveness to DMARD therapy, we observe a significant reduction in plasma MCTR3 concentrations in patients with erosive disease when compared to those that did not display signs of joint erosion (Figure S1).

**Table 1:** Summary of patient characteristics.

	Patients (n=99)
Pathotype (n)	Lymphoid (28), Fibroid (28), Myeloid (33), Ungraded (10)

Ethnicity	Asian (8), Bangladeshi (6), Bengali (1), Black (10), Black African (4), Black Caribbean (3), British (1), Caribbean (5), Caucasian (50), Chinese (1), Filipino (1), Indian (2), Korean (1), Mixed Greek (1), Not stated (2), Pakistani (1), Somalian (1), Sudanese (1)

Gender (n)	Female (67), Male (31)

Age at Recruitment – years	53 (±16)

Onset	6 (±3)

Currently smoking (%)	8 (8)

Co-Morbs (n)	Acne (2), Acute mi (1), Allergy to penicillin (1), Anaemia (2), Angina (1), Asthma (13), Axonal neuropathy (1), Basal cell carcinoma (1), Bladder cancer (1), Prostate cancer (1), Cardiovascular disease (1), Carpal tunnel syndrome (1), Cervical spondylitis (1), Chronic obstructive pulmonary disease (2), Coronary artery disease (1), Depression (4), Detrusor instability (1), Diabetes (1), Diverticulitis (1), Dyslipidoemia (1), Endometriosis (1), Enlarged prostate (2), Erectile dysfunction (1), Fatty liver (1), Fistula-in-ano (1), Foot & shoulder surgery (1), Gastric ulcers (1), Gastritis (1), Gastro-oesophageal reflux disease (2), Glaucoma (3), Gout (1), Graves disease (1), Hay fever (3), Heart surgery (1), Heart valve repaired (1), Hypercholesterolemia (14), Hypertension (40), Hysterectomy (2), Irritable bowel syndrome (2), Ischaemic heart disease (5), Kidney disease (1), Knee osteoarthritis (1), Lower back pain (2), Meniscus tear/knee (1), Menorrhagia (1), Multiple sclerosis (1), Osteoarthritis (5), Osteopenia (1), Osteoporosis (1), Peptic ulcer (1), Poor vision (1), Psoriasis (3), Reactive iritis (1), Rectal incontinence (1), Renal impairment (1), Rubello in utero (1), Scleritis (1), Shingles (1), Sickle cell trait (1), Sinusitis (1), Spina bifida occulta (1), Thalassaemia (1), Transient ischaemic attack (1), Type 1 diabetes (1), Type 2 diabetes (7), Varicose veins (1), Vitamin D deficiency (4)

Concomitant Med. (n)	Adcal (1), Adizem (1), Alendronate (1), Allopurinol (2), Aminophylline (1), Amitriptyline (6), Amlodipine (11), Antihypertensives (1), Arcoxia (2), Arthrotec (1), Aspirin (10), Atenolol (5), Atorvastatin (3), Bendroflumethiazide (5), Bisoprolol (1), Budesonide (2), Budromide (1), Ca vitamin (8), Ca-antagonist (1), CaD3 (1), Calci-chew (1), Candesartan (4), Carbimazole (1), Celecoxib (1), Cetirizine (1), Citalopram (1), Clenil mod (1), Clopidogrel (2), Co-codamol (15), Codeine (1), Co-dydramo (2), Co-tenidone (1), D Vitamin (9), Detrusitol, Diclofenac (8), Dihydrocodeine (3), Dipyridamole (1), Docusate (1), Doxazosin (3), Etoricoxib (2), Ferrous sulphate (5), Finasteride (1), Flixotide (1), Fluoxetine (3), Fluticasone (1), Formoterol (1), Furosemide (4), Gabapentin (1), Gaviscon (1), Gliclazide (1), Glimepiride (1), Glucosamin (1), GTN (3), HRT (1), Ibuprofen (13), Indapamide (1), inhaler (asthma), Insulin 3), Irbesartan (2), Isosorbide (2), Ivabradine (1), Lacrilube (1), Lansoprazole (8), Lantus (1), Latanoprost (1), Levothyroxine (4), Lipitor, Lisinopril (3), Losartan (2), Lyrica (1), Metformin (8), Movicol (1), Multivitam (1), Naproxen (10), Nicorandil (1), NSAIDs (7), Omeprazole (5), Oromorph (1), Paracetamol (7), Peppermint (1), Perindopril, (2), Phyllocont (1), Piogitazon (1), Piroxicam (1), Prednisolone (2), Pregabalin (2), Quinine su (1), Ramipril (8), Repaglinid (1), Salbutamol (6), Senokot (1), Seretide (2), Sibicol (1), Simvastatin (16), Slidenafil (1), Solgar sup (1), Spiriva (1), Symbicort (1), Talisartan (1), Tamsulosin (3), Temazepam (1), Tetracycline (2), Thyroxine (4), Timolol (2), Tiotropium (1), Tramadol (6), Trimethoprim (1), Ventolin (2), Voltarol (1), Zolair (1)

Recent Steroid Therapy	No (50), Yes (13)

Steroid Treatment	Depo-Medro (7), Fluticasone (1), Prednisone (5)

DMARD Treatment (n)	ASA (2), ASA/HCQ (3), HCQ (3), MTX (4), MTX/ASA (46), MTX/ASA/HCQ (9), MTX/ASA/HCQ/LEF (1), MTX/HCQ (17), MTX/HCQ/LEF (1),

ESR	36 (±29)

CRP	19 (±30)

CCP	215 (±224)

RF	104 (±163)

Tiredness VAS	42 (±30)

Pain VAS	55 (±29)

Pt. VAS Global Health	65 (±26)

Physician VAS Global Assess	65 (±23)

Tender Joints	13 (±8)

Swollen Joints	8 (±6)

HAQ	1.5 (±0.7)

DAS28	5.8 (±1.3)

**Table 2:** Correlation between peripheral blood MCTR concentrations and disease activity in DMARD naive RA patients.

	MCTR1	MCTR2	MCTR3
ESR	r = -0.1111; (CI = -0.308 to 0.952); p = 0.276	r = -0.099; (CI = -297 to 0.107); p =0.3323	r = -0.254; (CI = -0.435 to -0.0523) **p = 0.012**
CRP	r = -0.058; (CI = -261 to 0.151); p = 0.578	r = -0.227; (CI = -0.414 to -0.022); **p = 0.026**	r = -0.315; (CI = -0.499 to -0104); **p = 0.003**
DAS28	r = 0.025; (CI = 178 to 0.227); p = 0.803	r = -0.177; (CI = -0.367 to 0.274); p = 0.080	r = -0.337; (CI = -0.505 to -0.144); **p = 0.0001**

Plasma was collected from a patient cohort of DMARD naive patients (n= 99 patients) and concentrations for MCTR1, MCTR2 and MCTR3 were established using lipid mediator profiling (see methods for details). (Concentrations for each of these meditators were then correlated with DAS28 scores as well as plasma C-reactive protein (CRP) and erythrocyte sedimentation rate (ESR) using Spearman correlation, where bold was used to represent significant p values).

### MCTR3 displays anti-arthritic activity in inflammatory arthritis

Having observed a significant relationship between MCTR3 concentrations and disease activity we next questioned whether pharmacological administration of MCTR3 would modulate joint disease progression and severity in experimental arthritis. For this purpose, we employed a serum transfer model of inflammatory arthritis, which relies on the activation of the innate immune system replicating the effector phase of rheumatoid arthritis.^46^ Administration of MCTR3, obtained *via* stereoselective total organic synthesis^28^ (see Figure S2 for physical characterization), immediately after disease onset conferred protection against joint inflammation as observed by a significant reduction in clinical scores and improvements in histological markers of disease. This included a decrease in leukocyte infiltration, and increased safranin staining, a measure of glycosaminoglycan content in the cartilage (Figure S3a-c). Given the roles that lipid mediators have in both propagation (e.g. prostaglandins and leukotrienes) and resolution of joint inflammation (e.g. SPM) we next evaluated whether MCTR3 administration also regulated the joint lipid mediator profile. Using partial least square discriminant analysis, which produces a regression model built using concentrations of lipid mediators differently expressed between the two groups, we found a shift in joint lipid mediator concentrations in mice treated with MCTR3. This shift was linked with a downregulation of pro-inflammatory and nociceptive eicosanoids including PGE_2_ and PGF_2a_ and an upregulation of pro-resolving and anti-nociceptive mediators such as MaR1 and PDX (Figure S3d,e and Table S4).

**Figure 1:**
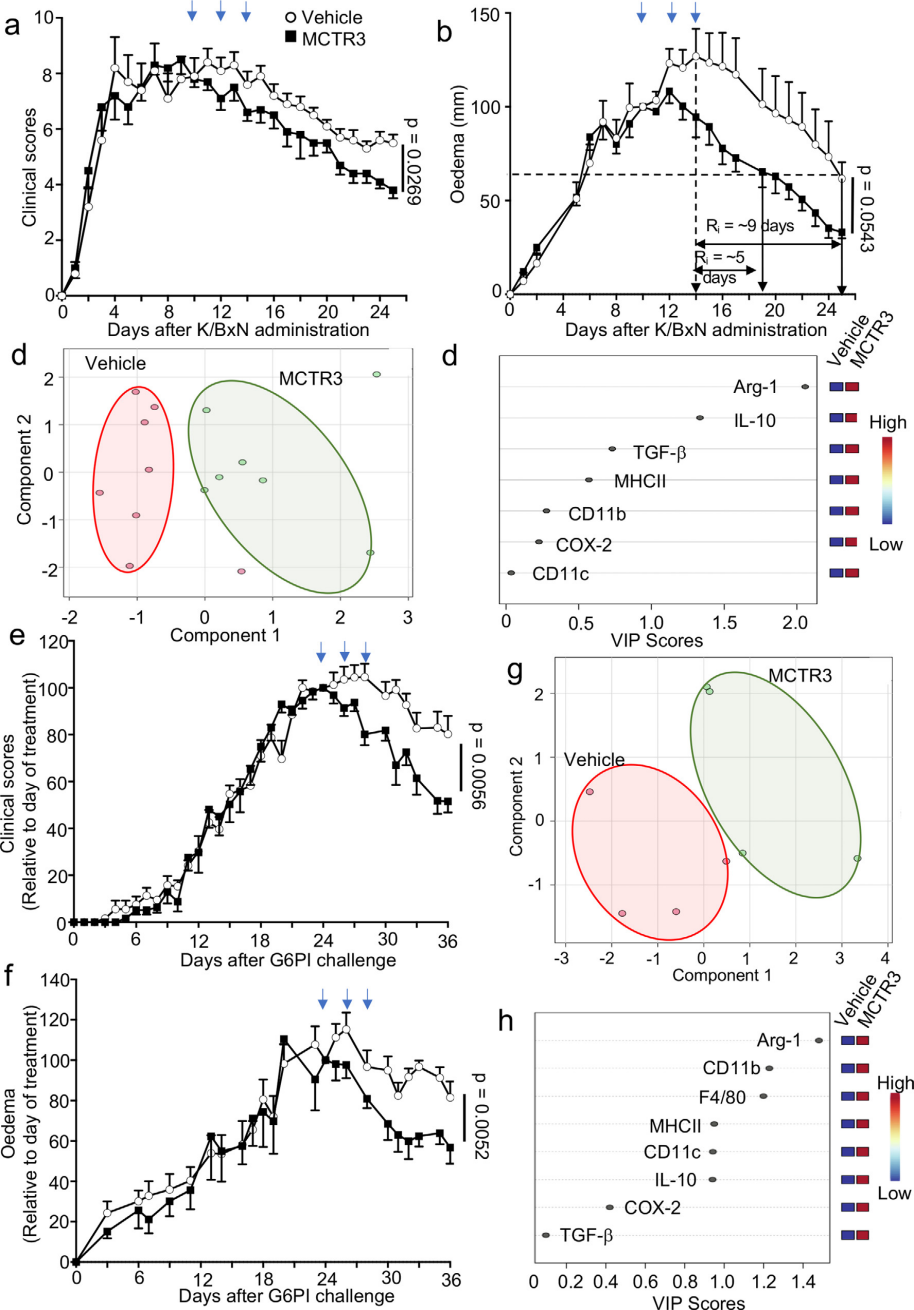
MCTR3 displays anti-arthritic activity in inflammatory arthritis. (a-d) K/BxN serum (100 µL *via intraperitoneal* injection, i.p.) was administered to C57BL/6 mice on days 0, 2 and 8 to initiate and prolong inflammatory arthritis. Mice were treated with 1 µg/mouse MCTR3 or vehicle (DPBS + 0.1 % EtOH) on day 10, 12 and 14 *via intravenous* (i.v.) injection. Disease course was assessed by evaluating (a) clinical scores and (b) paw oedema daily. Results are mean ± SEM, n = 9 mice per group. (Statistical differences were evaluated using a Two-Way ANOVA). Arrows denote days when MCTR3 was administered. (c, d) paws were harvested on day 25, cells liberated from the joints and the expression of phenotypic markers was evaluated on joint macrophages using flow cytometry and PLS-DA. (d) Scores plot with highlighted regions denoting the clusters representing cells from each group and (d) VIP scores for each of the markers evaluated. Each dot in the score plot represents a separate mouse. (e-h) Arthritis was induced in DBA/1 mice by administration of 10 µg G6PI in CFA on day 0. On day 24, 26 and 28 (denoted in blue arrows), mice were administered 1 µg/mouse MCTR3 or vehicle (DPBS + 0.1 % EtOH) i.v. and (e) clinical scores and (f) paw oedema were measured. Results are mean ± SEM, n = mice 7 per group from 2 separate experiments. (Statistical differences were evaluated using a Two-Way ANOVA). (g-h) paws were harvested on day 36, cells liberated from the joints and the expression of phenotypic markers was evaluated on joint macrophages using flow cytometry and PLS-DA. (f) Scores plot with highlighted regions denoting the clusters representing cells from each group and (h) VIP scores for each of the markers evaluated. Each dot in the score plot represents a separate mouse.

We next tested whether MCTR3 also displayed joint protective activities when administrated later in the disease course. For this purpose, we used a model of sustained joint inflammation.^47^ MCTR3 was administered 10 days after disease onset and joint inflammation was evaluated throughout the disease course. Here we found that treatment of mice with MCTR3 accelerated the resolution of joint inflammation as demonstrated by a shortening of the resolution interval from ∼9 days to ∼5 days, a significant reduction in clinical scores and a marked reduction in joint oedema (Figure 1a,b). Histological evaluation of joints collected from these mice demonstrated that MCTR3 reduced all the parameters evaluated, significantly reducing both leukocyte infiltration and cartilage damage (Figure S4).

Mononuclear phagocytes, in particular MDM, play a central role in the propagation and termination of inflammation,^10^^,^^12^ as well as tissue repair and regeneration.^48^^,^^49^ Therefore, we next evaluated whether MCTR3 governed MDM phenotype in arthritic joints. Flow-cytometric evaluation of phenotypic markers in cells isolated from mice treated with MCTR3 demonstrated a marked shift in phenotype as highlighted by a shift in the cluster representing cells obtained from these mice when compared with cells isolated from mice treated with vehicle alone (Figure 1c, Figure S5). To evaluate which of the phenotypic markers were responsible for this shift in macrophage markers we evaluated the Variable in Importance (VIP) scores, whereby a VIP score >1 identifies those variables that contribute to the observed separation between the two groups. This demonstrated that the shift in phenotype was primarily linked with the upregulation of two markers in cells from MCTR3 treated mice, namely Arginase (Arg)-1 and Interleukin (IL)-10 (Figure 1d).

We next assessed whether the protective activities of MCTR3 were retained in a model of adaptive immune system-driven arthritis using the glucose-6-phosphate isomerase peptide driven model of inflammatory arthritis.^50^ Here, administration of MCTR3 during the course of arthritic inflammation also led to a reduction in joint inflammation as measured by a decrease in both clinical scores and joint oedema (Figure 1e,f). Notably, the MDM-directed activities of MCTR3 were retained in this model, as demonstrated by the marked shift in the macrophage phenotype observed in cells isolated from joints of MCTR3 treated mice when compared with those isolated from joints of vehicle treated mice (Figure 1g). This shift in phenotype was linked with an upregulation of three phenotypic markers, including Arg-1 and CD11b, in cells obtained from joints of MCTR3 treated mice (Figure 1h). Taken together these findings suggest that MCTR3 treatment alters joint MDM phenotype and reduces arthritic inflammation.

### MCTR3 promotes bone and cartilage repair

**Figure 2:**
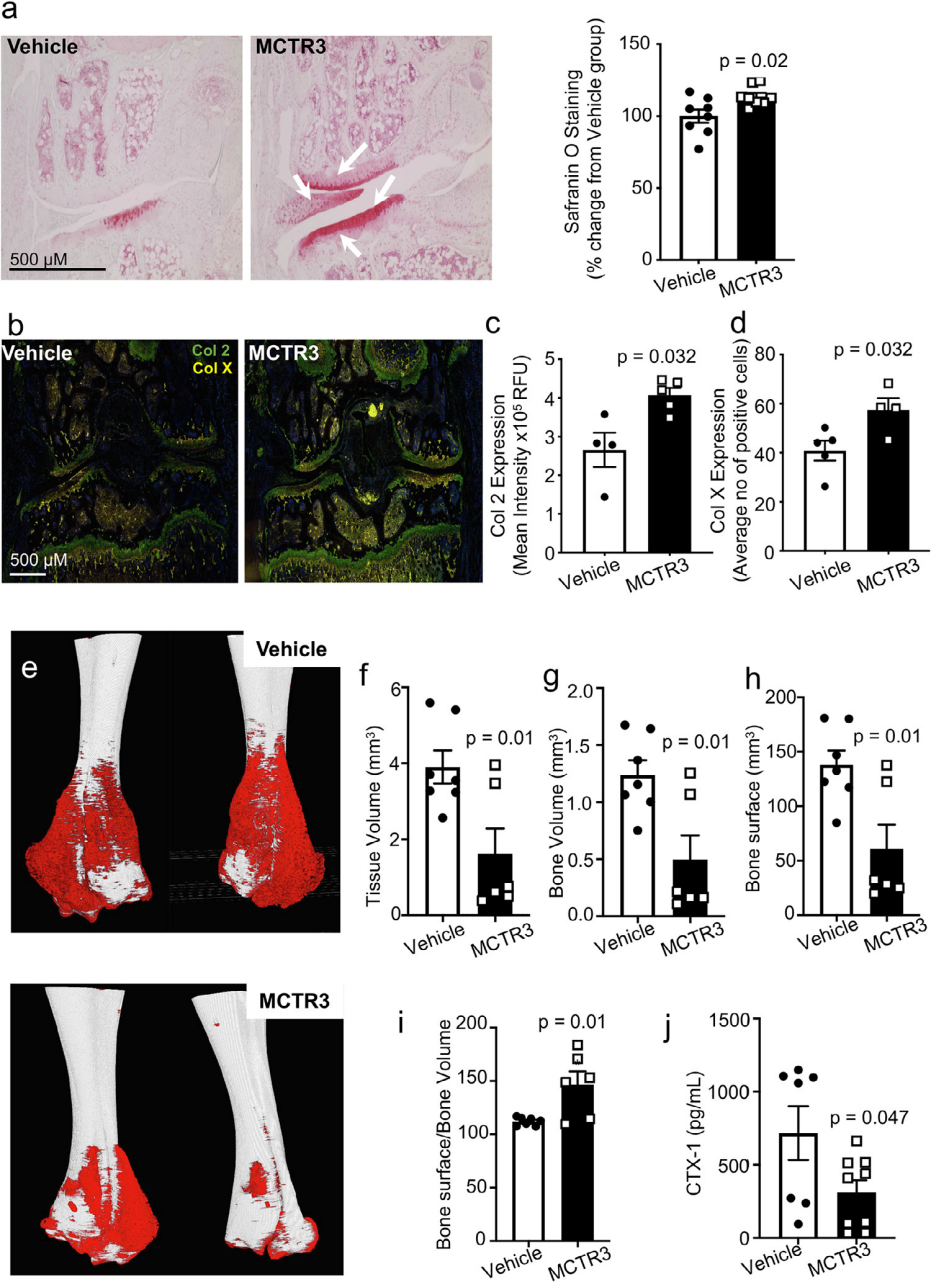
MCTR3 promotes bone and cartilage repair. C57BL/6 mice were administered 100 µL K/BxN serum *i.p.* on day 0, 2 and 8. Mice were treated with 1 µg/mouse MCTR3 or vehicle (DPBS + 0.1 % EtOH) *i.v.* on day 10, 12 and 14. Hind paws and blood were collected on day 25 and (a) glycosaminoglycan content was evaluated using Safranin-O staining. (*left and centre panels*) Representative images from Safranin-O stained knee joints and (*right panel*) quantification of Safranin-O stained knee joints. Results are mean ± SEM, n = 7-8 per group. (Statistical differences were evaluated using Mann-Whitney U test). (b-d) Expression of collagen type 2 (Col 2) and collagen type X (Col X) was evaluated using immunohistochemistry. (b) Representative images from immunofluorescent assessment of Col 2 and Col X expression, (c) mean intensity of Col 2 and (d) average number of cells expressing Col X. Results are mean ± SEM, n = 4-5 per group, statistical differences were evaluated using Mann-Whitney U test. (e-i) microCT analysis was performed on ankle joints *ex vivo* to assess bone callus cover. (e) Representative images of ankles from vehicle and MCTR3 treated mice, where red represents the callus area. (f) Callus tissue volume, (g) bone volume, (h) bone surface and (i) the ratio of bone surface to bone volume of the ankles from mice treated with either vehicle or MCTR3. Results are mean ± SEM, n = 6-7 per group from two separate experiments. (Statistical differences were evaluated Mann-Whitney U test). (j) Blood was collected at the end of the experiment and plasma CTX-I levels were assessed. Results are mean ± SEM, n = 7 for vehicle and n = 9 for MCTR3 groups. (Statistical differences were evaluated Mann Whitney U test).

Chronic inflammation in RA is associated with both cartilage and bone degradation which is the main cause of debilitation in patients with RA.^5^ Thus, we next questioned whether MCTR3, in addition to reducing joint inflammation and cartilage damage also promoted joint repair. To address this question, we investigated whether MCTR3 regulated cartilage repair in arthritic mice. For this purpose, we used safranin O-staining to evaluate glycosaminoglycan content in joints from arthritic mice. Here we observed higher safranin O-staining in joints from MCTR3 treated mice when compared with vehicle treated mice in both mice challenged with K/BxN serum and G6PI peptide (Figure 2a and Figure S6a, b). Furthermore, immunohistochemical staining of joints from MCTR3 treated mice demonstrated an increase in the expression of both collagen 2, the principal molecular component in mammalian cartilage,^51^ and that of collagen X, which is expressed in the calcified zone of cartilage that interfaces with bone^51^ in mice challenged with K/BxN serum and treated with MCTR3 (Figure 2b-d). Thus, these results demonstrate that MCTR3 displays cartilage-protective activities in inflammatory arthritis.

Since one of the main debilitating features of arthritis is rapid bone remodelling, we next determined whether the joint protective actions of MCTR3 extended to the bone. We first evaluated whether MCTR3 treatment in K/BxN induced arthritis limited pathological bone formation by evaluating callus cover in long bones from arthritic mice. Analysis using microCT demonstrated smaller bone calluses on MCTR3 treated mice, as measured by the assessment of the total volume and surface area occupied by the callus (Figure 2e-h). Notably, these changes were linked with a significant increase in overall surface to volume ratio in the callus, a marker of callus mineralization and therefore bone integrity (Figure 2i). This decrease in callus size was linked with a reduction in plasma concentrations of C-terminal telopeptide of type 1 collagen (CTX-1), a marker of bone resorption, in MCTR3 treated mice (Figure 2j). Thereby these findings suggest that MCTR3 limits bone damage in inflammatory arthritis.

Given that MCTR3 displayed joint protective activities in the G6PI model of chronic arthritic inflammation which is linked with marked bone resorption^52^ we next queried whether MCTR3 was also able to reverse this process. Here, we used microCT analysis to investigate bone volume in arthritic joints, comparing bone volumes on day 24, prior to MCTR3 treatment, to those at day 35. This analysis demonstrated that bone volume in vehicle treated arthritic mice was reduced, in line with the sustained disease activity (Figure 1e,f). Whereas, bone volume in MCTR3 treated mice was increased (Figure S6c, d). These findings indicate that MCTR3 reduces the increased bone and cartilage turnover characteristic of arthritic inflammation, thereby suggesting that this autacoid activates protective mechanisms to improve both cartilage and bone integrity in inflammatory arthritis.

### MCTR3 reprograms monocyte responses to reduce inflammation and repair inflamed joints

**Figure 3:**
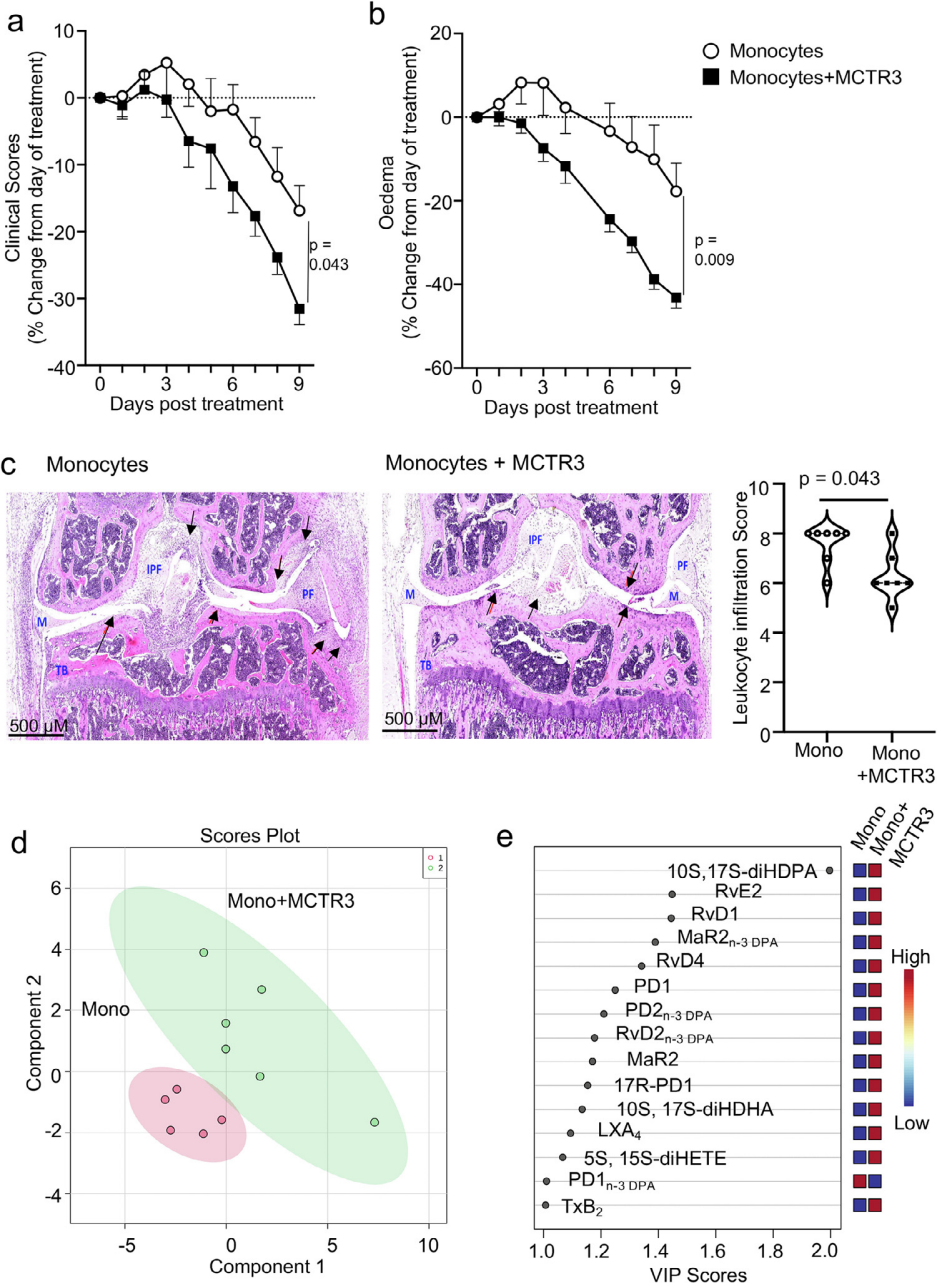
MCTR3 reprograms monocyte responses to reduce inflammation and repair inflamed joints. (a-b) K/BxN serum (100 µL, *i.p.*) was administered to C57BL/6 mice on day 0, 2 and 9 to induce and prolong inflammatory arthritis and, on day 12, mice were treated *i.v.* with 2×10^6^ monocytes isolated from arthritic mice and incubated with either vehicle (DPBS + 0.1 % EtOH) or 1 nM MCTR3 for 90 min at 37°C. Disease course was monitored daily by assessing (a) clinical scores and (b) oedema. Results are mean ± SEM and expressed as percent change from day of treatment. n = 9 per group from two distinct experiments. (Statistical differences were evaluated using a Two-Way ANOVA). (c) On day 22 hind paws were harvested joints were fixed, sectioned, stained using H&E stain and leukocyte infiltration evaluated. *Left and centre panels* present representative images from each experimental group; *right panel* Quantitation of the scores in each of the group. Results are mean ± SEM. n = 7 mice per group. (Statistical differences were evaluated using Mann-Whitney U test). IFP = intrapatellar fat, M = meniscus, TB = Tibia, PF = Pannus formation, arrows denote leukocyte infiltration. (d-e) Paws were harvested 10 days after treatment and lipid mediator profiles were determined using LC-MS/MS-based lipid mediator profiling and evaluated using PLS-DA. (d) scores plot with highlighted regions denoting the clusters representing cells from each group and (e) VIP scores for top 15 mediators. Each dot in the score plot represents a separate mouse.

Having observed that MCTR3 treatment led to a shift in MDM phenotype, a reduction in disease severity and increased joint repair, we next queried whether the joint protective actions of MCTR3 were linked with the reprogramming of circulating monocytes to yield MDM with pro-resolving and tissue reparative properties. To test this hypothesis, we evaluated whether *ex vivo* treated monocytes from arthritic mice recapitulated the joint protective activities of MCTR3. Here, we incubated bone-marrow derived monocytes from donor arthritic mice with 1 nM of MCTR3 (MCTR3-reprogrammed monocytes) or vehicle and after 90 minutes cells were washed and administered to arthritic mice. This concentration was selected since we previously observed that it optimally regulated both tissue regeneration in planaria and macrophage efferocytosis.^22^ We then treated mice with 1×10^6^ monocytes, a dose that was selected based on published literature demonstrating the ability of mononuclear phagocytes to influence disease activity.^53^ In mice administered the MCTR3-reprogrammed monocytes we observed a reduction in disease severity, as demonstrated by a significant reduction in clinical scores and oedema, when compared with mice treated with monocytes incubated with vehicle alone (Figure 3a, b). The protective activities exerted by MCTR3-reprogrammed monocytes were observed at a histological level, where H&E staining revealed a significant reduction in leukocyte infiltration into the inflamed paws in mice treated with MCTR3-reprogrammed monocytes (Figure 3c). We also observed that MCTR3-reprogrammed monocytes regulated joint lipid mediator concentrations as observed by a shift in the cluster representing lipid mediator profiles obtained from joints of mice treated with these cells compared with mice treated with monocytes incubated with vehicle only (Figure 3d). Notably, assessment of the top 15 mediators differentially regulated between the two groups demonstrated a marked upregulation of several joint protective SPM, including RvD1, in paws from mice receiving MCTR3-reprogrammed monocytes suggesting that these cells exert potent pro-resolving activities (Figure 3e, Table S5).

**Figure 4:**
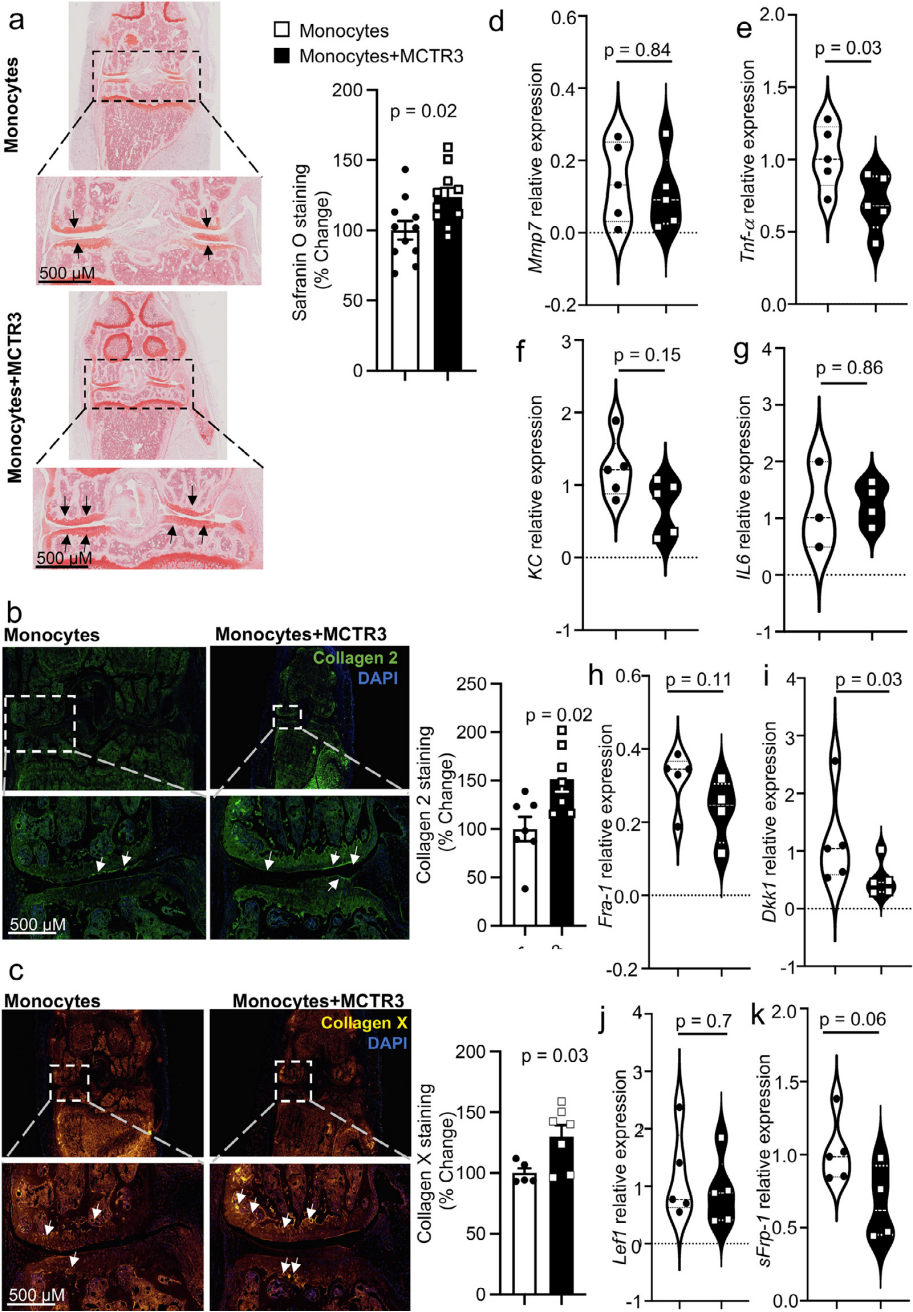
Regulation of joint reparative pathways in mice receiving MCTR3-reprogrammed monocytes. K/BxN serum (100 µL, *i.p.*) was administered to C57BL/6 mice on day 0, 2 and 9 to induce and prolong inflammatory arthritis and, on day 12, mice were treated *i.v.* with 2×10^6^ monocytes isolated from arthritic mice and incubated with either vehicle (DPBS + 0.1 % EtOH) or 1 nM MCTR3 for 90 min. (a-c) On day 22 hind paws were harvested, fixed, and stained to evaluate A) proteoglycan content using Safranin-O staining; (b) Col 2 and (c) Col X expression was evaluated using immunofluorescence. *Left panels* present representative images from each experimental group, *right panels* provide a quantitative evaluation of the staining. Results are mean ± SEM and expressed as percent change vs Monocyte group. n = 7-8 mice per group. (Statistical differences were evaluated using Mann Whitney-U test). (d-k) Hind paws were also collected on day 22 to evaluate the expression of the indicated genes using quantitative realtime PCR. Results are from n = 3-5 mice per group. (Statistical differences were evaluated using Mann Whitney-U test).

We next evaluated whether MCTR3-reprogrammned monocytes regulated tissue repair in arthritic mice. For this purpose, we assessed glycosaminoglycan content using Safranin-O staining in articular cartilage. This analysis demonstrated a significant increase in Safranin-O staining in mice treated with MCTR3-reprogrammed monocytes when compared with mice that were treated with monocytes alone (Figure 4a). This increase in cartilage cover was linked with a significant increase in the expression of collagen 2 and collagen X in mice treated with MCTR3-reprogrammed monocytes (Figure 4b,c).

To evaluate the mechanisms that lead to both the reduction in inflammatory arthritis and the upregulation of reparative mechanisms, we next evaluated the expression of molecules known to be involved in the propagation of inflammation and in the regulation of joint repair. We first evaluated the expression of KC, the murine homologue of IL-8, IL-6, Tumour necrosis factor (TNF)-α, matrix metalloproteinase (MMP) 7 and Fos-related antigen (Fra)-1.^54^ While *Mmp7* and *IL-6* expression in arthritic paws from both groups was essentially similar, the expression of *KC, Tnf-α* and *Fra-1* was decreased in arthritic paws from mice treated with MCTR3-reprogrammed monocytes, reaching statistical significance for *Tnf-α* (Figure 4d-h)*.* Having observed a significant regulation of *Tnf-α* in mice receiving the reprogrammed monocytes, we next evaluated the expression of downstream targets of TNF-α which are known to regulate the Wnt signalling pathway, a key pathway in both bone and cartilage maintenance.^55^^,^^56^ For this purpose, we assessed the expression of Dickkopf (Dkk)-1, Lymphoid Enhancer Factor (LEF)-1,^57^ and Secreted frizzled-related protein (sFRP)-1.^58^ This analysis demonstrated that while *Lef-1* and *sFrp-1* expression was essentially similar between the two groups, *Dkk-1* expression was significantly downregulated in mice treated with MCTR3-reprogrammed monocytes when compared with mice receiving monocytes alone (Figure 4i-k). Together these findings indicate that MCTR3-reprogrammed monocytes activate reparative mechanisms linked with both bone and cartilage repair.

### MCTR3 reprograms the arthritic monocyte-derived macrophage transcriptome

We next sought to determine the mechanism(s) by which MCTR3 elicited its protective actions. We first evaluated whether MCTR3 reprogramming modulated the ability of these cells to migrate and/or be retained in the arthritic joints. For this purpose, we labelled monocytes with the cell membrane-binding fluorescent dye PKH67 prior to reinjection into arthritic mice and then evaluated their recruitment into the hind leg joints of arthritic mice using flow cytometry. This analysis demonstrated that ∼2% of the total number of monocytes administered were recoverable from these tissues after 10 days. Notably, there was no significant difference in the relative abundance of PKH67+ macrophages found in these tissues between the two groups. These findings suggest that despite the transferred monocytes representing a relatively small subset of total cells in the arthritic joints they display potent anti-inflammatory and reparative activities. They also suggest that MCTR3-reprogramming does not influence the ability of monocytes to migrate and/or differentiate into macrophages within the arthritic joints and therefore the observed differences are likely to arise from a change in the phenotype of these cells.

**Figure 5:**
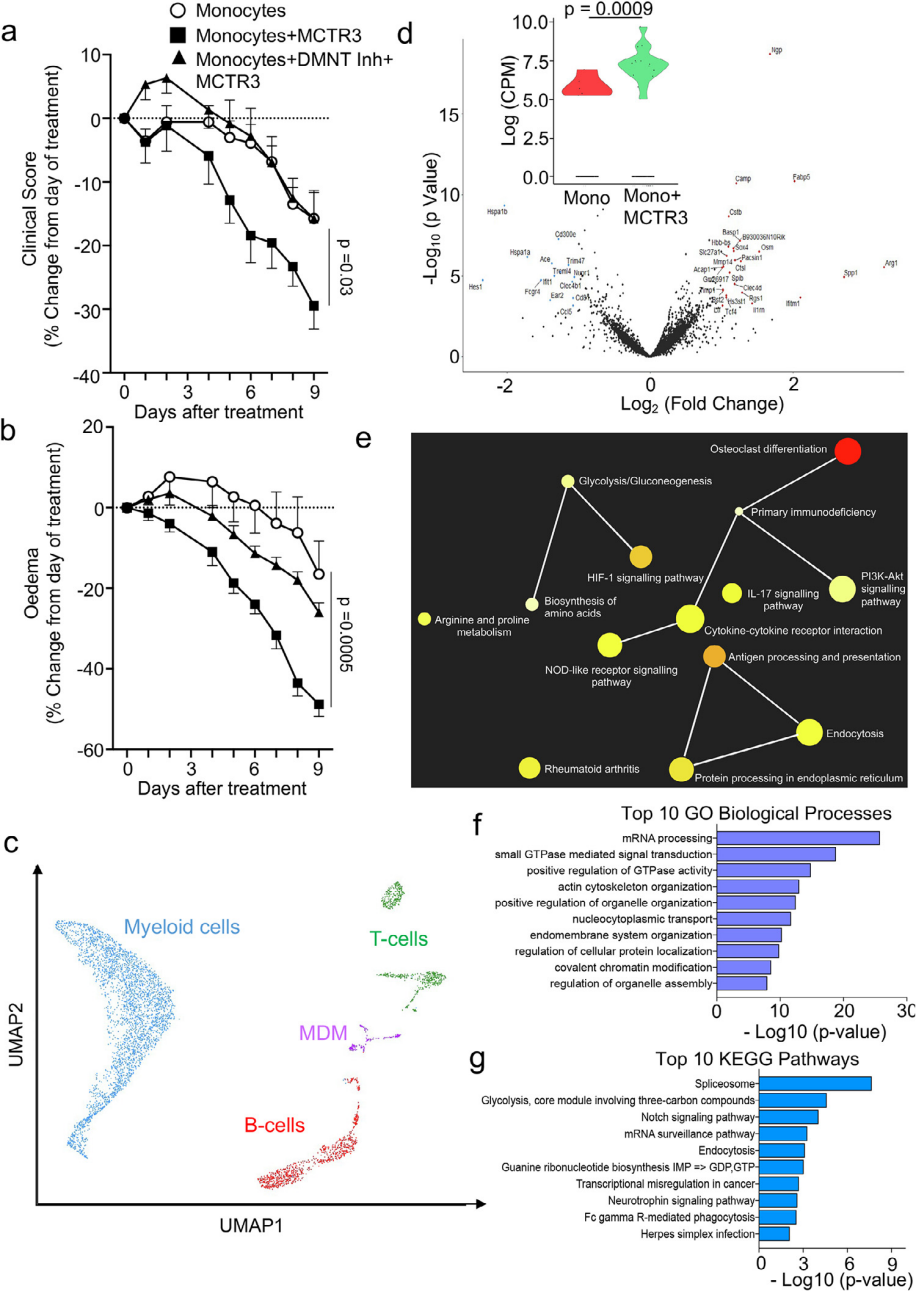
MCTR3 reprograms the arthritic monocyte-derive macrophage transcriptome. (a-b) Arthritis was induced and prolonged in C57BL/6 mice by administering 100 µL K/BxN serum i.p. on day 0, 2 and 9. Mice were treated on day 12 *i.v.* with 2×10^6^ monocytes isolated from arthritic mice and incubated either with vehicle (DPBS + 0.1 % DMSO) or 10 µM RG108, a DNMT inhibitor, for 15 min and then with a vehicle (DPBS + 0.1 % EtOH) or 1 nM MCTR3 for 90 min (37 °C). Disease course was monitored daily by assessing (a) clinical scores and (b) oedema. Results are mean ± SEM and expressed as percent change from day of treatment. n = 10 mice per group. (Statistical differences were evaluated using Two-way ANOVA). (c-e) K/BxN serum (100 µL, *i.p*.) was administered to mice on days 0, 2 and 9 to induce and prolong inflammatory arthritis. On day 12, mice were treated *i.v.* with 2×10^6^ monocytes isolated from arthritic mice that were previously incubated with either vehicle (DPBS + 0.1 % EtOH) or 1 nM MCTR3 for 90 min. Cells were isolated from paw joints on day 22, sorted for CD45^+^ cells and single cell RNA sequencing was performed. c) UMAP plots of clusters obtained from isolated leukocyte populations, (d) volcano plot highlighted differentially regulated genes in the MDM population (*inset*) relative gene regulation for Arg-1. (Statistical differences were evaluated using Mann Whitney test). (e) The gene network analysis for genes that were found to be differentially regulated in MDM from mice receiving MCTR3-reprogrammed monocytes when compared with MDM receiving monocytes incubated with vehicle. Results are from n = 4 mice per group. (f-g) Monocytes were isolated from human healthy volunteers, these cells were then incubated with GM-CSF (7 days, 37°C) then with either Vehicle or MCTR3 (1nM, 37°C). Cells were lysed and the phosphoproteome determined using mass spectrometry. (f) GO Biological pathway analysis and (g) KEGG pathway analysis for proteins found to be differentially phosphorylated in cells incubated with MCTR3 when compared to those incubated with vehicle. Results are representative of cells from n = 3 healthy volunteers per group.

Recent studies demonstrate that changes in the epigenetic landscape of innate immune cells, including monocytes and macrophages, leads to their long term reprograming.^59^ Having observed that short term incubation of monocytes with MCTR3 led to long term protective actions in arthritis we next queried whether this was at least in part linked with the regulation of the epigenetic landscape of the cells. Given the central role that DNA methyltransferases^59^^,^^60^ play in this process we next tested whether inhibition of these enzymes would reverse the protective activities of MCTR3-reprogrammed monocytes. Indeed, while disease severity was significantly reduced in mice administered MCTR3-reprogrammed monocytes, incubation of these cells with a DNA methyltransferase inhibitor (RG108), abolished the protective actions of MCTR3 as observed by a decrease in the ability of these cells to regulate joint inflammation (Figure 5a,b). Thus, these findings indicate that MCTR3 regulates circulating monocyte responses to limit joint inflammation and promote joint repair in a DNA methyltransferase-dependent manner.

To further explore the mechanism activated in MCTR3-reprogrammed monocytes that contribute to the observed protective actions, we next incubated monocytes with MCTR3 as detailed above and administered them to arthritic mice. After 10 days we collected joints, sorted the leukocytes and subjected these cells to single cell RNA sequencing. Assessment of transcript expression differences between cells isolated from mice receiving MCTR3-reprogrammed monocytes and those from mice receiving monocytes incubated with vehicle demonstrated that of the different cell subsets identified, the biggest changes in transcript levels were observed in MDM with 41 differentially regulated genes (Figure 5c,d, Figure S7 and Table S6). Notably, out of these differentially expressed genes, Arginase-1 (Arg-1) was the gene that was upregulated to the greatest extent in MDM isolated from mice receiving MCTR3-reprogrammed monocytes (Figure 5d). Network analysis of genes that were found to be differentially regulated in MDM from MCTR3-reprogrammed monocytes demonstrated a differential regulation of genes linked with several processes involved in joint repair, including osteoclast differentiation and arginine and proline metabolism (Figure 5e).

We next evaluated the signaling pathways activated by MCTR3 using a phosphoproteomic approach. Gene ontology analysis of proteins found to be differentially phosphorylated in mononuclear phagocytes incubated with MCTR3 versus those incubated with vehicle alone demonstrated a marked regulation of proteins involved in post-transcriptional regulation and protein translation by MCTR3 (Figure 5f and Table S7). This regulation was also observed when using the Kyoto Encyclopaedia of Genes and Genomes pathway database that highlighted an enrichment of spliceosome linked proteins as well as proteins involved in mRNA surveillance by MCTR3 (Figure 5g and Table S7). In these studies, we also found that MCTR3 regulated the phosphorylation status of several proteins involved in both epigenetic and chromatin modification, including that of methylases Histone-lysine N-methyltransferase SETD2 as well as the deacetylases Histone deacetylase 1 and Histone deacetylase 2 (Table S7).

Recent studies identified several macrophage subsets in arthritic joints that are linked with different aspects of disease onset/propagation and resolution of joint disease.^14^^,^^61^ Therefore, we evaluated whether the transcriptional changes observed in macrophages from mice receiving MCTR3-reprogrammed monocyte were linked with the reprogramming of a specific macrophage population. Using the taxonomy published by Culemann and colleagues^61^ we identified three macrophage populations in our single cell RNAseq dataset. These were MHCII^+^ Interstitial macrophages, RELM-a^+^ Interstitial macrophages, and CX_3_CR1^+^ lining macrophages, with the latter cells being the most abundant macrophage population in this dataset (Figure S8a). Assessment of the overall number of genes found to be differentially regulated in each of these subsets demonstrated that the largest changes in gene expression were in CX_3_CR1^+^ lining macrophages. These included the upregulation of Arg-1 in cells isolated from arthritic joints of mice that received MCTR3-reprogrammed monocytes (Figure S8b-e). Taken together these findings indicate that MCTR3 promotes the reprograming of mononuclear phagocytes *via* the regulation of epigenetic programs and protein translation to facilitate the termination of inflammation and joint protection. They also support the hypothesis that MCTR3-reprogrammed monocytes recruited into arthritic joints exert their activities *via* the upregulation of tissue protective pathways.

**Figure 6:**
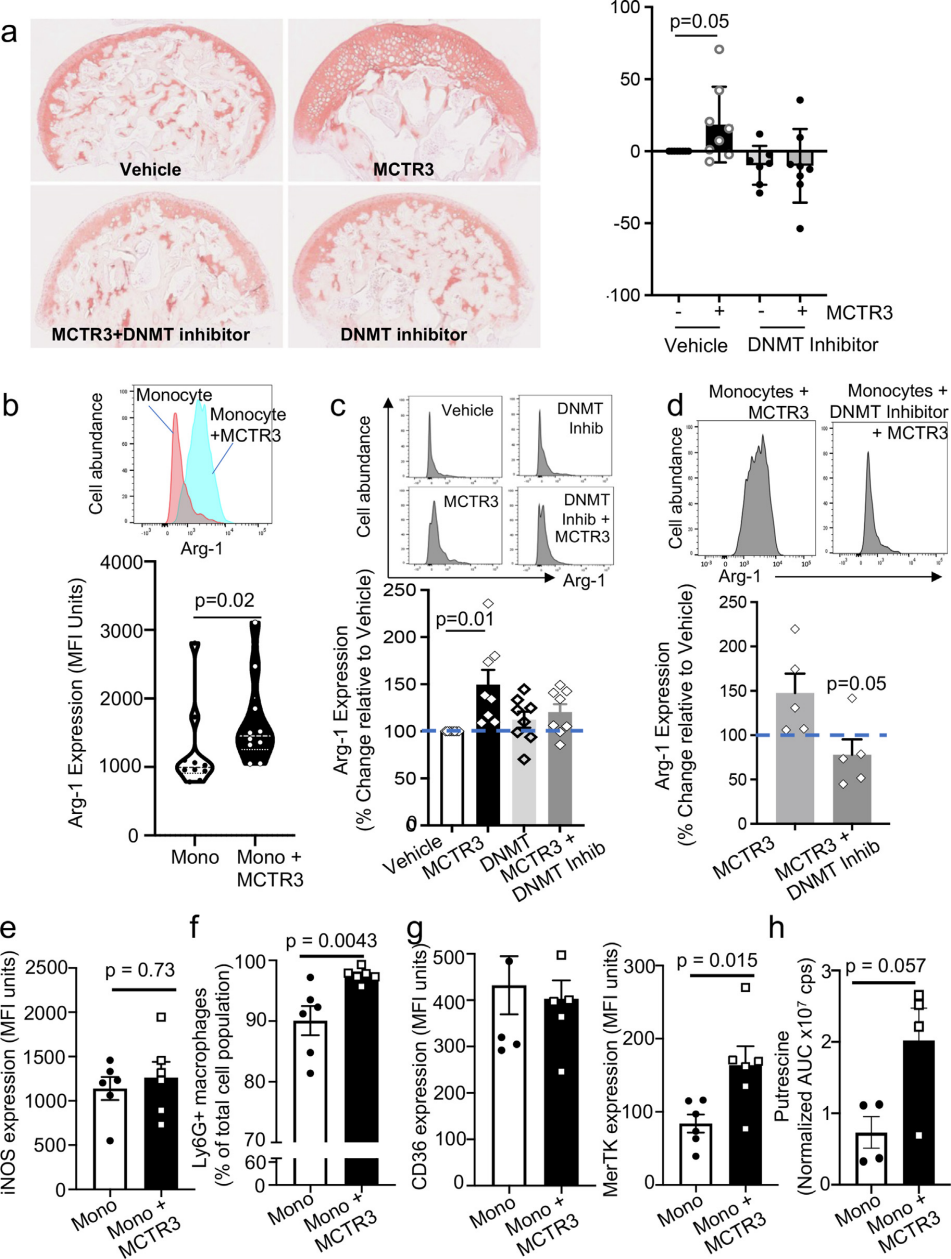
DNMT enzyme inhibition reverses the ability of MCTR3 to upregulate Arg-1 in MDM and the cartilage protective activities of MCTR3-reprogrammed monocytes.. (a) Inflammatory arthritis was induced in C57BL/6 mice by administering 100 µL K/BxN serum i.p. on days 0 and 2 and femur heads and bone marrow monocytes were collected on day 5. Femur heads were incubated in serum free DMEM high glucose containing 1% insulin-transferrin-selenium for 48 hours and then in DMEM containing 10 % FBS and 10 ng/mL IL1-β for a further 72 hours. Bone marrow derived monocytes were incubated with vehicle (DPBS + 0.1 % DMSO) or 10 µM RG108, a DNMT inhibitor, for 45 min and then with either vehicle (DPBS + 0.1 % EtOH) or 1 nM MCTR3 for 24 hours. Cells were differentiated to monocyte-derived macrophages, then incubated with femur heads for 48 hours. The proteoglycan concentrations in the femur heads were assessed using Safranin O staining. (*left panels*) Representative images from Safranin-O stained femur heads (*right panel*) quantification of Safranin-O stained femur heads. Results are mean ± SEM and expressed as percentage change from cells incubated with vehicle alone. n = 5-8 mice per group from two separate experiments. (Statistical differences were evaluated using one-sample Wilcoxon signed rank test). (b) Mice were administered K/BxN serum on days 0, 2 and 9 then on day 12 they were treated 2×10^6^ PKH67-labelled monocytes isolated from arthritic mice and incubated with either vehicle (DPBS + 0.1 % EtOH) or 1 nM MCTR3 for 90 min *via i.v* injection. After 10 days joints were harvested, cells were liberated and expression of Arg-1 was evaluated in PKH67^+^CD64^+^ cells using flow cytometry. Results are from n=10 mice per group. (Statistical differences were evaluated using a Mann-Whitney U test). (c) Inflammatory arthritis was induced in C57BL/6 mice and bone marrow-derived monocytes were isolated and treated as in a and Arg-1 expression was evaluated using flow cytometry. Results are mean ± SEM and expressed as percentage change from cells incubated with vehicle alone. n = 8 per group from two separate experiments. (Statistical differences were evaluated using one-sample Wilcoxon signed rank test.) (d) Mice were given K/BxN serum (*via i.p*. injection) on days 0, 2 and 9. On day 12 these were treated with 2×10^6^ PKH67-labelled monocytes isolated from arthritic mice and incubated either with vehicle (DPBS + 0.1 % DMSO) or 10 µM RG108, a DNMT inhibitor, for 15 min and then with a vehicle (DPBS + 0.1 % EtOH) or 1 nM MCTR3 for 90 min. On day 22, joints were collected and the expression of Arg-1 in PKH67^+^ CD64^+^ cells was evaluated using flow cytometry. Dashed line represents vehicle groups. Results are mean ± SEM and expressed as a percentage change from vehicle group. (Statistical differences were calculated using a Mann-Whitney U test). (e-h) Mice were treated as described above in (b) and cells liberated from the hind paw on day 22 were evaluated for (e) Expression of iNOS, (f) Ly6G (g) CD36 and MerTK expression in macrophages. (h) Putrescine levels in paws. Results are mean ± SEM and expressed as percentage change from vehicle group. n = 4-6 mice per group. (Statistics differences were evaluated Mann-Whitney U test).

To evaluate this hypothesis further, we employed an organ culture system whereby monocytes were obtained from the bone marrows of arthritic mice and incubated with or without MCTR3 in the presence or absence of a DNA methyltransferrase inhibitor. These cells where then differentiated to macrophages for 5 days using GM-CSF as the agonist given that this was recently linked with macrophage differentiation in joints from RA patients.^62^ We then incubated arthritic femur heads with these cells for 2 days and assessed their glycosaminoglycan expression. Assessment of Safranin-O staining demonstrated higher glycosaminoglycan content in femur heads incubated with MDM obtained from MCTR3-reprogrammed monocytes (Figure 6a). Of note, inhibition of methyltransferase activity reversed these protective actions of MCTR3-reprogrammed monocytes (Figure 6a). Thereby, these findings lend support to the hypothesis that MCTR3 reprograms monocyte responses resulting in MDM that display joint protective activities.

### Arg-1 mediates the joint protective activities of MCTR3-reprogrammed monocytes

Recent studies demonstrate that Arg-1 exerts potent joint protective activities in inflammatory arthritis.^54^^,^^63^ Having observed an upregulation of Arg-1 in joint macrophages from mice treated with MCTR3 (Figure 1) and those treated with MCTR3-reprogrammed monocytes (Figure 5) we next questioned whether Arg-1 was responsible for the observed anti-arthritic properties of these cells. For this purpose, we repeated the *in vivo* experiments detailed above, this time labelling the monocytes isolated from arthritic mice with a fluorescent membrane dye, PKH67, to differentiate them from endogenous monocytes. After 10 days we harvested the paws, liberated cells and assessed the expression of Arg-1 in PKH67^+^ MDM. Here we observed a significant increase in Arg-1 expression in PKH67^+^ MDM from mice that received MCTR3-reprogrammed monocytes when compared with those that received monocytes incubated with vehicle alone (Figure 6b). This observation was also replicated in MDM differentiated *in vitro* from MCTR3-reprogrammed monocytes and previously found to exert cartilage protective activities in our organ culture system (Figure 6c). Notably, incubation of monocytes with a methyltransferase inhibitor reversed the ability of MCTR3 to upregulate this joint protective enzyme both *in vitro* and *in vivo* (Figure 6c,d). Intriguingly, the regulation of Arg-1 was found to be a specific response elicited by MCTR3 in these monocytes, given that iNOS expression in these cells, an M1-lineage associated marker, was unaffected by MCTR3 (Figure 6e).

Efferocytosis is a key step in the resolution of inflammation and is linked with initiation of reparative responses.^21^^,^^64^ Therefore, we next evaluated whether MCTR3 reprogramming enhanced the ability of joint macrophage to upregulate the clearance of apoptotic cells. Flow cytometric analysis demonstrated a significant increase in the levels of macrophages carrying apoptotic neutrophils, as shown by an increase in the number of macrophages expressing the neutrophil marker Ly6G (Figure 6f). These results were coupled with a significant increase in the expression of the efferocytosis receptor MerTK (Figure 6g). Since we observed an increase in both Arg-1 and MerTK we next queried whether the downstream product of Arg-1, putrescine, which is linked with the regulation of MerTK expression and efferocytosis in macrophages,^65^ was also increased in mice receiving MCTR3-reprogrammed monocytes. Mass spectrometric analysis demonstrated an upregulation of putrescine in joints from mice receiving MCTR3-reprogrammed monocytes when compared with those that received monocytes incubated with vehicle alone, although this increase did not reach statistical significance (Figure 6h).

**Figure 7:**
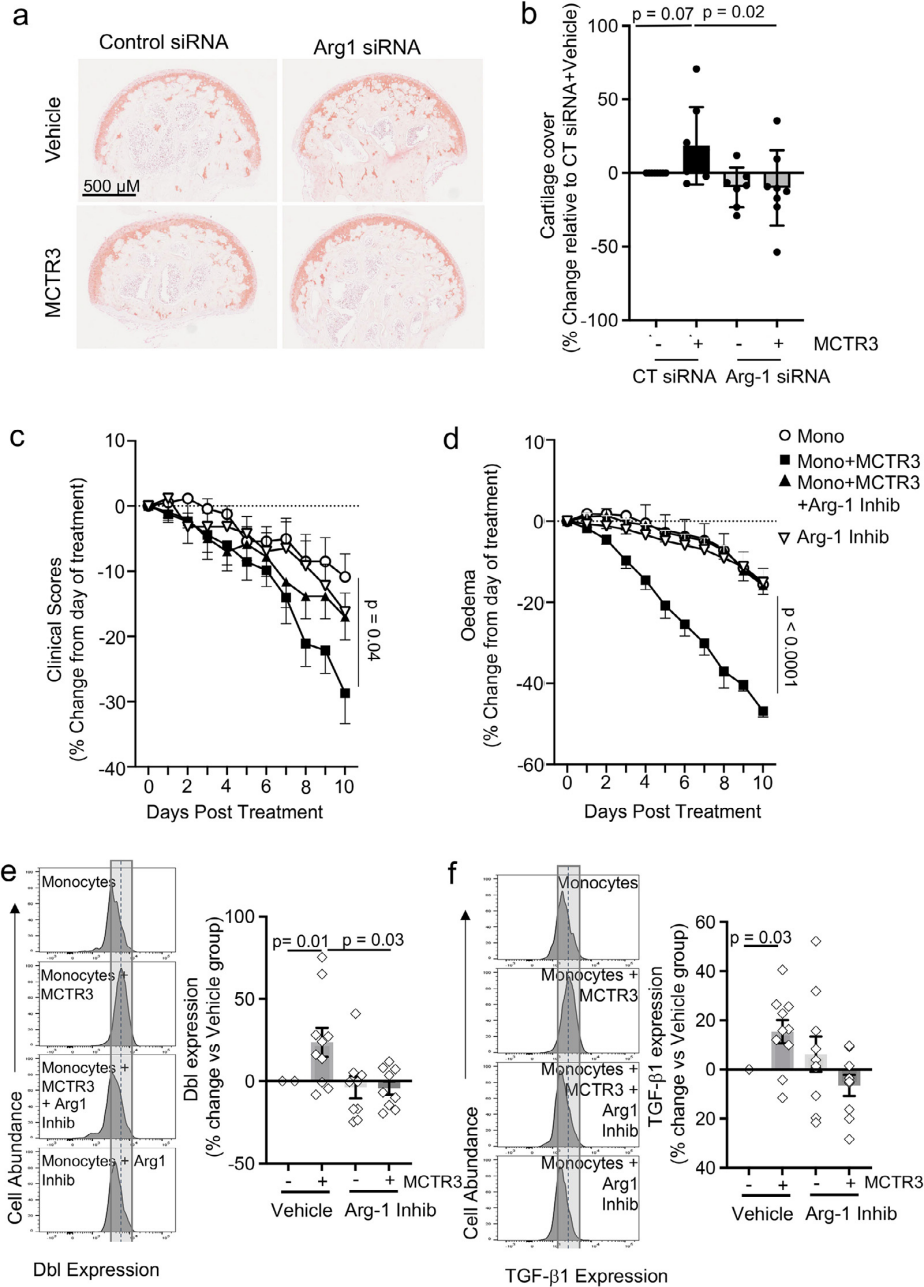
Arg-1 mediates the anti-inflammatory and cartilage protective activities of MCTR3-reprogrammed monocytes. (a, b) Femur heads and bone marrow-derived monocytes were collected 5 days following the induction of arthritis in C57BL/6 mice. Femur heads were incubated in serum free DMEM high glucose containing 1% insulin-transferrin-selenium for 48 hours and then in DMEM containing 10 % FBS and 10 ng/mL IL-1β for 4 days. Monocytes were incubated with vehicle (DPBS + 0.1 % EtOH) or 1 nM MCTR3 for 24 hours, then differentiated to monocyte-derived macrophages. Two days after the initiation of differentiation, cells were transfected with control siRNA or siRNA against Arg-1. Three days later these cells were incubated with arthritic femur heads for 48 hours. Tissues were then collected and glycosaminoglycan content was evaluated using Safranin-O staining. (a) Representative images and (b) quantitation of Safranin-O staining. Results are mean ± SEM, n = 7-8 mice per group. (Statistical differences were evaluated using one sample Wilcoxon signed rank test when assessing for differences vs Vehicle group and using one-way ANOVA and Kruskal Wallis *post hoc* test when evaluating differences vs MCTR3 treated group). (c-f) Mice were administered K/BxN serum on days 0, 2 and 9 then on day 12 they were treated 2×10^6^ PKH67-labelled monocytes isolated from arthritic mice and incubated with either vehicle (DPBS + 0.1 % EtOH) or 1 nM MCTR3 for 90 min *via i.v* injection and 200 µg N^ω^-Hydroxy-nor-L-arginine (nor-NOHA), an arginase 1 inhibitor, or vehicle (DPBS) that were administered daily for a 10-day period *via i.p.* injection. Disease course was evaluated by assessing (c) clinical scores and (d) paw oedema. Results are mean ± SEM, n = 8-10 per group from two distinct experiments and expressed as percent change from first day of treatment. (Statistical differences were evaluated using Two-way ANOVA). (e-f) At the end of the experiments joints were collected and (e) Dbl and (f) TGF-β1 expression was evaluated in PKH67^+^ CD64^+^ cells using flow cytometry. Results are mean ± SEM and expressed as percentage change from vehicle group. n = 9-10 mice per group. (Statistical differences were evaluated using one-sample Wilcoxon signed rank test when assessing for differences vs Vehicle group and using one-way ANOVA and Kruskal Wallis *post hoc* test when evaluating differences vs MCTR3 treated group).

To further investigate the role of Arg-1 in mediating the antiarthritic and reparative activities of MCTR3-reprogrammed monocytes we used an siRNA approach to knockdown the expression of this enzyme in MDM and then evaluated the ability of these cells to promote cartilage repair using the organ culture system described above. Here we found that while MDM obtained from MCTR3-reprogrammed monocytes and transfected with a control siRNA markedly increased glycosaminoglycan content in arthritic femur heads, transfection of cells with siRNA to Arg-1, significantly abrogated the cartilage protective actions of these cells (Figure 7a,b).

We next tested whether inhibition of Arg-1 activity *in vivo* would also reverse the protective actions of MCTR3-reprogrammed monocytes. For this purpose, we treated mice that received MCTR3-reprogrammed monocytes with the Arg-1 inhibitor N^ω^-hydroxy-nor-ʟ-arginine (nor-NOHA) and assessed joint disease activity. Here we found that as observed in previous experiments, administration of MCTR3-reprogrammed monocytes led to a reduction in both clinical scores and joint oedema, when compared with those mice that received monocytes incubated with vehicle alone. Of note treatment of mice with the Arg-1 inhibitor reversed these joint protective actions of MCTR3-reprogrammed monocytes as observed by an increase in clinical scores and oedema (Figure 7c-d). Furthermore, inhibition of Arg-1 activity also reversed the upregulation of the Rac1 guanosine triphosphate (GTP)-exchange factor Dbl, an enzyme implicated in mediating the pro-resolving actions of Arg-1 in MDM^65^ and TGF-β1, a morphogen involved in bone and cartilage maintenance and repair (Figure 7e, f).^66^ Together these findings support a role for Arg-1 in mediating the joint protective activities of MCTR3-reprogrammed monocytes.

## Discussion

Despite the notion that joint damage in patients with RA leads to significant morbidity, current therapeutic approaches in RA are ineffective at activating joint repair programs. In the present studies we found that MCTR3 concentrations were negatively correlated with markers of both systemic and joint inflammation in patients with RA. Systemic administration of MCTR3 to arthritic mice not only accelerated the resolution of joint inflammation but also activated joint reparative programs. Assessment of the cellular mechanisms involved in the observed protective activities demonstrated that MCTR3 reprogrammed monocytes upregulated a number of tissue protective mechanisms, including Arg-1 expression. Intravenous administration of MCTR3-reprogrammed monocytes recapitulated the protective activities of systemic MCTR3 administration. Inhibition of DNA methyltransferases or Arg-1 abrogated both the anti-inflammatory and joint protective actions of MCTR3 during inflammatory arthritis. Together these findings suggest, that MCTR3 reprograms circulating monocytes that when recruited into inflamed joints, differentiate into macrophages with potent anti-inflammatory and tissue reparative activities.

Unremitting inflammation is a key component in the destruction of joint tissues. In many patients this leads to severe deformation of the articular bones. Whilst such deformations in large articular bones, such as hips and knees can be rectified *via* arthroscopic surgery, smaller joints such as those in the fingers cannot be rectified using these approaches resulting is severe disability in many patients with RA.^1^, ^2^, ^3^, ^4^, ^5^^,^^48^^,^^67^ To date, the only therapeutics that impinge on this process of joint destruction are anti-TNF therapies which have been found to limit the activation of synovial cells and the progression of tissue destruction.^3^^,^^4^ Nonetheless, these therapeutics do not activate reparative process, and therefore any damage that occurs, especially in those patients with advanced bone and joint destruction are likely to be permanent. Furthermore, not all patients treated with anti-TNF therapies go into remission, which results in further tissue damage. In the present studies we found that MCTR3 administration, using a therapeutic paradigm, potently limited clinical signs of joint inflammation in two distinct models of inflammatory arthritis. This reduction in joint inflammation was linked with the activation of joint protective mechanisms as demonstrated by a significant upregulation in both collagen 2 and collagen X expression, increased cartilage cover, increased bone volume and decreased callus size in joints from mice treated with MCTR3. The anti-inflammatory activities of MCTR3 are also in line with observations made with other SPMs, whereby AT-RvD1, RvD3, MaR1 and the RvD precursor 17-HDHA were all found to display potent anti-inflammatory, anti-nociceptive and joint protective activities.^27^^,^^40^^,^^68^^,^^69^

Trained immunity is now appreciated to play a significant role in both host protection from pathogenic infections as well as in the propagation of inflammation in chronic inflammatory conditions.^70^ Underpinning trained immunity is a change in the DNA methylation status of the cell that leads to a shift in the cellular responses to subsequent inflammatory stimulus. Studies investigating this process in RA demonstrated that circulating CD14+ monocytes from these patients expressed increased basal CD11b expression and produced higher concentrations of IL-1β and IL-6 when stimulated *ex vivo*.^71^ Notably, recent findings demonstrated that incubation of human monocytes with etanercept and adalimumab downregulated the trimethylation of H3K4, H3K27, H3K36 and H3K79 in the CCL2 promoter region by decreasing the expression of the related methyltransferases WDR5 and Smyd2.^72^ These finding suggest therapeutics that can modulate trained immunity to downregulate the expression of inflammation-initiating genes in monocytes and MDMs may be attractive targets in the treatment of RA.

Notably, the process of trained immunity has to-date been primarily linked with the reprogramming of cells towards an activated, potentially pro-inflammatory status.^59^^,^^71^ Our findings indicated that MCTR3-reprogrammed monocytes exert both anti-inflammatory and tissue reparative activities as observed by a decrease in joint disease activity and upregulation in collagen 2 and collagen X expression. These protective activities of MCTR3 on reprogramming monocyte responses were reversed when these cells were incubated with a DNA methyltransferase inhibitor, underscoring a central role for epigenetic reprogramming in mediating the protective activities of MCTR3 on monocytes. Thus, the present findings suggest that MCTR3 changes the epigenetic landscape of trained monocytes from arthritic mice to upregulate tissue protective and pro-resolving pathways in these cells. This hypothesis is supported by findings made in our transcriptomic and phosphor-proteomic analysis. Whereby we found that MCTR3 regulates the phosphorylation status of proteins involved in epigenetic and chromatin regulation in mononuclear phagocytes. Furthermore, sc-RNA seq analysis of synovial leukocytes from mice receiving MCTR3-reprogrammed monocytes demonstrated a marked shift in the transcriptome of MDMs with an upregulation of several immunoregulatory and host protective genes, including Arg-1. This enzyme was recently found to exert anti-arthritic activities, whereby upregulation of Arg-1 expression in macrophages in mice lacking the transcription factor Fra-1, which negatively regulates Arg-1 expression, was linked with an enhanced resistance to both joint inflammation and joint damage in experimental arthritis.^54^ Arg-1 activity in macrophages is also involved in regulating efferocytosis, a key pro-resolving activity of macrophages which was recently suggested to play an important role in tissue repair.^73^ In this context, the Arg-1 downstream metabolite putrescine regulates the ability of macrophages to uptake apoptotic cells, and disruptions in this pathway are linked with the propagation of tissue inflammation in atherosclerosis.^65^ In the present studies we found that MCTR3-reprogrammed monocytes yield MDMs with elevated expression of Arg-1 both *in vivo* and *in vitro*. This observation was linked with a downregulation of *Fra-1* expression in arthritic paws from these mice. Notably, inhibition of Arg-1 expression or activity reversed both the anti-inflammatory and the joint reparative activities of MCTR3-reprogrammed monocytes. Thus, these findings establish a central role of this enzyme in mediating the protective activities of MCTR3 during inflammatory arthritis.

Over the past years there has been a steady increase in the development of cell-based therapeutics for the treatment of both chronic inflammatory conditions and cancers. Many of these therapies involve complex and sometimes expensive gene editing approaches which have to some extent limited their application. In addition, these approaches have been primarily focused on the use of bone marrow-derived cells. Harvest of these cells is a painful process for patients, it requires hospitalization, and can cause side effects that include bone marrow inflammation further complicating the application of cell-based therapies. Another approach being developed is that of allogeneic cell therapies, which would in principle increase throughput and drive down costs. However, the application of this approach to patient treatment has been fraught with issues primarily linked with the activation of the recipient immune responses and rejection. Results from the present studies indicate that circulating monocytes may represent a target population for cell-based therapeutics in patients with RA. A recent study supports the potential applicability of such an approach, whereby, Moroni and colleagues in a phase I study collected peripheral blood monocytes from patients with liver cirrhosis. They then differentiated the cells to MDMs *ex vivo* and re-injected these cells back to the patient, demonstrating that such an approach is both feasible and safe.^74^ Our findings from both *in vivo* studies and organ culture models suggest that MCTR3-reprogrammed monocytes display enhanced protective activities over non-reprogrammed monocytes in facilitating both the resolution of joint inflammation and promoting the repair and regeneration of arthritic tissues.

The present work leverages well established experimental methodologies for dissecting and evaluating mechanisms in tissue repair therefore they establish insights into the potential utility of MCTR3 and MCTR3-reprogrammed monocytes in both regulating inflammation and promoting joint repair. Given that the evidence collected thus far is primarily based on experimental models, future studies will need to establish the translational potential of the present work to humans. They will also need to establish key pharmacological parameters as well as the targets that these potential SPM-based therapeutics may regulate.

In summation, the present studies demonstrate that MCTR3 reprograms monocytes to differentiate to MDMs with anti-inflammatory and joint reparative properties. Whilst results from the present findings do not exclude a role for other cells in mediating the protective activities of MCTR3 when administered *in vivo*, they provide compelling evidence for a central role of MDMs in conferring both the anti-inflammatory and joint protective activities of this mediator. The results presented herein suggest that the protection observed in mice receiving MCTR3-reprogrammed monocytes is mediated directly by the reprogrammed cells *via* the release of factors and/or molecules that exert joint reparative activities such as polyamines. The anti-arthritic propoertes of these cells are also likely propagated *via* the release of molecules, such as SPM, that reprogram the tissue environment and resolve inflammation (Figure 5-7). Together these mechanisms lead to the reduction of inflammation and repair of damaged tissues. Thus, our findings elucidate potential therapeutic strategies in the treatment of patients with RA that target both the inflammatory component and promote joint repair. This approach may also be useful in other inflammatory conditions characterized by uncontrolled inflammation and tissue destruction.

## Contributors

J.D. conceptualized the overall research plan, obtained funding, wrote the original draft of the manuscript and supervised the work; K.P., L.L., P.S, E.A.G, D.K., J.F. M.H., V.R. curated the data, performed formal analysis, J.S., A.R.R., B.W.S., A.P., C.P. J.D. supervised the work and/or provided resources. All authors contributed to reviewing and editing the manuscript. Data verification was carried out by J.D. and A.P. All authors read and approved the final version of the manuscript.

## Data Sharing statement

All data will be made available by the corresponding author upon reasonable request

## Declaration of interests

JD is an inventor on patents related to the composition of matter and/or use of pro-resolving mediators some of which are licensed by Brigham and Women's Hospital or Queen Mary University of London for clinical development. JD is also scientific founder and director of Resolomics Ltd. K.P. and L.L. are inventors on patent applications related to the use of cell therapy relating the use of MCTR3 reprogrammed monocytes to treat joint inflammation. Other authors declare no relevant conflict of interest.

## Appendix Supplementary materials

Image, application 1

Image, application 2
