# Supplementary figures and images for: MCTR3 reprograms arthritic monocytes to upregulate Arginase-1 and exert pro-resolving and tissue-protective functions in experimental arthritis
Source: eBioMedicine. 2022 Apr 14;79:103974. doi: 10.1016/j.ebiom.2022.103974 (PMC9038546; doi:10.1016/j.ebiom.2022.103974)

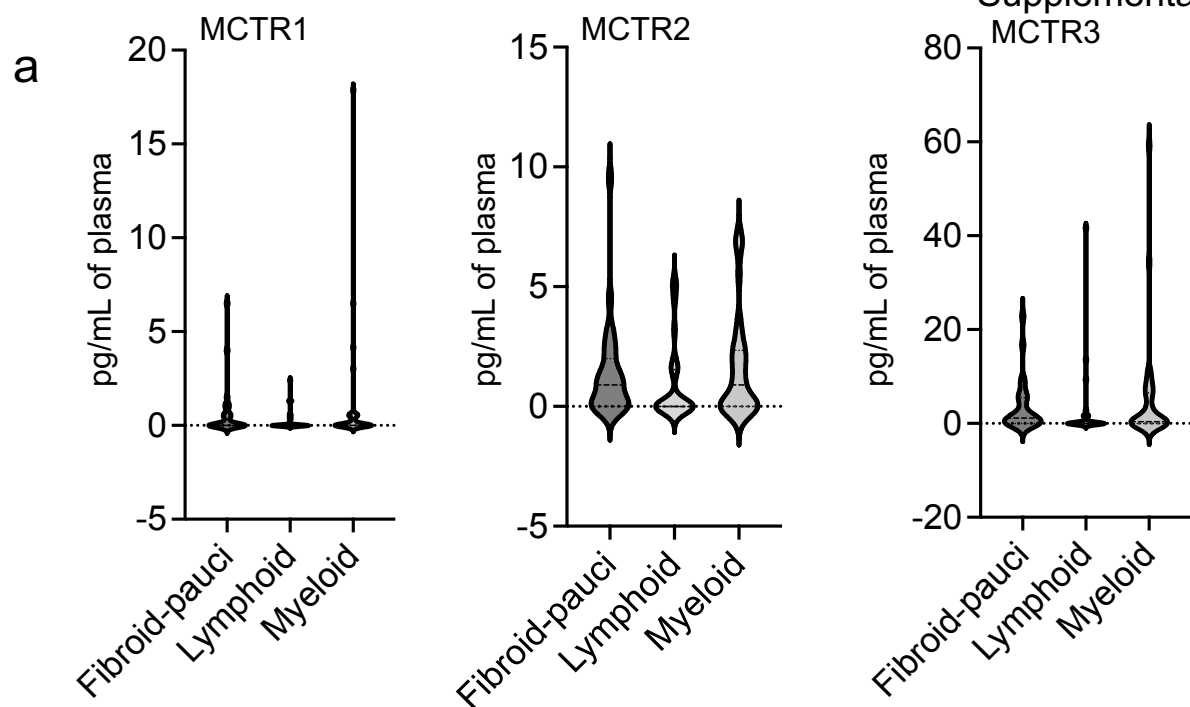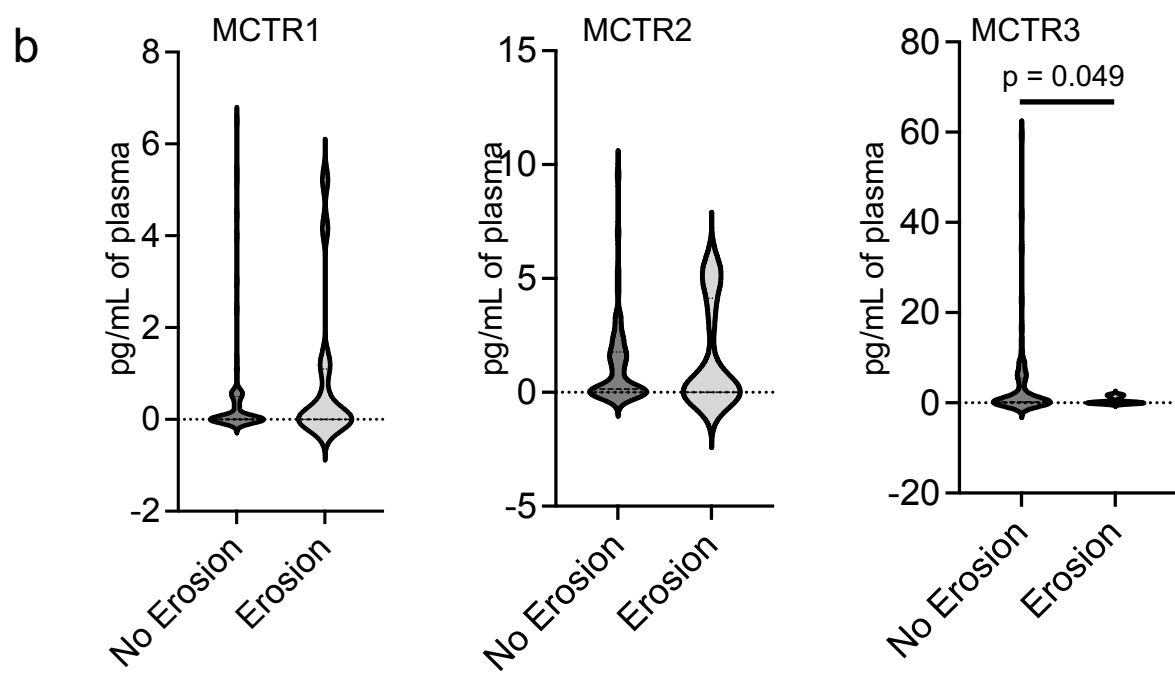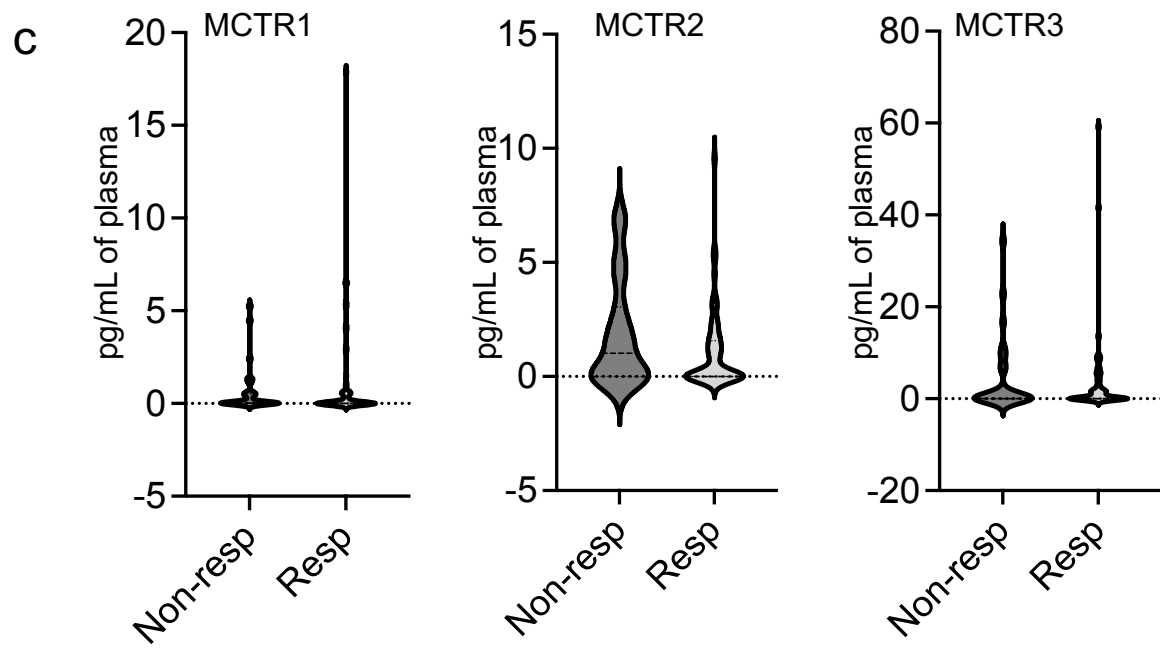

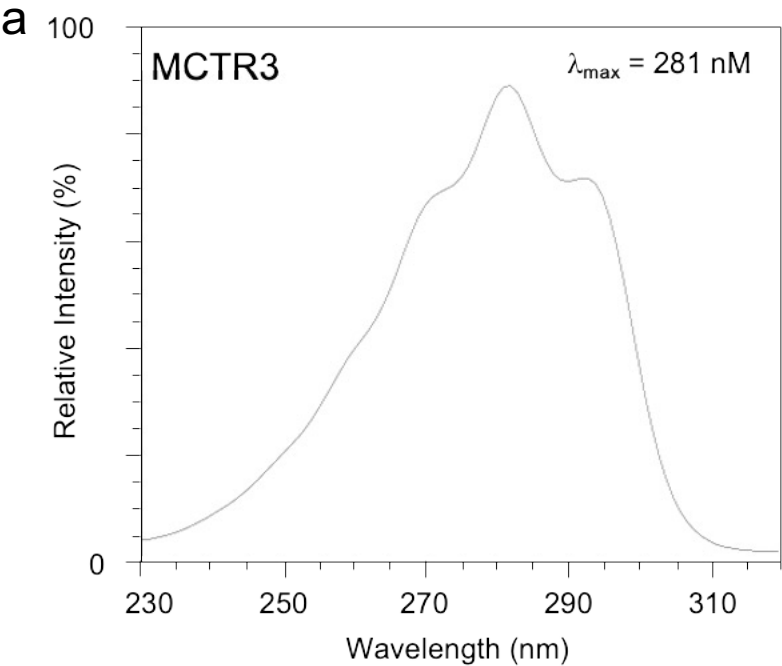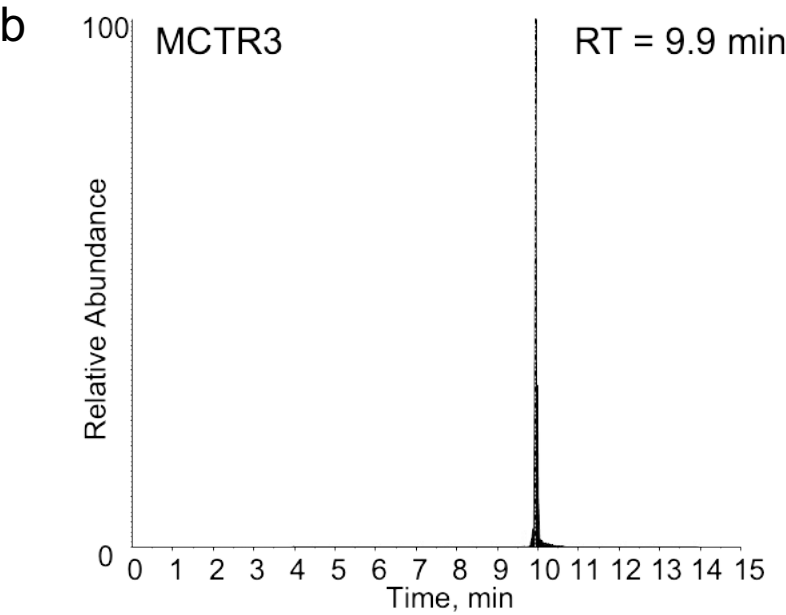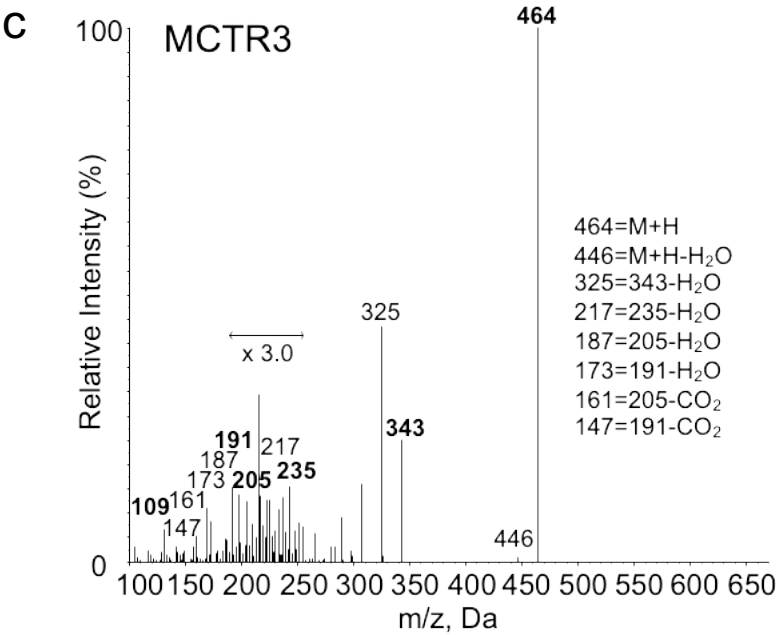

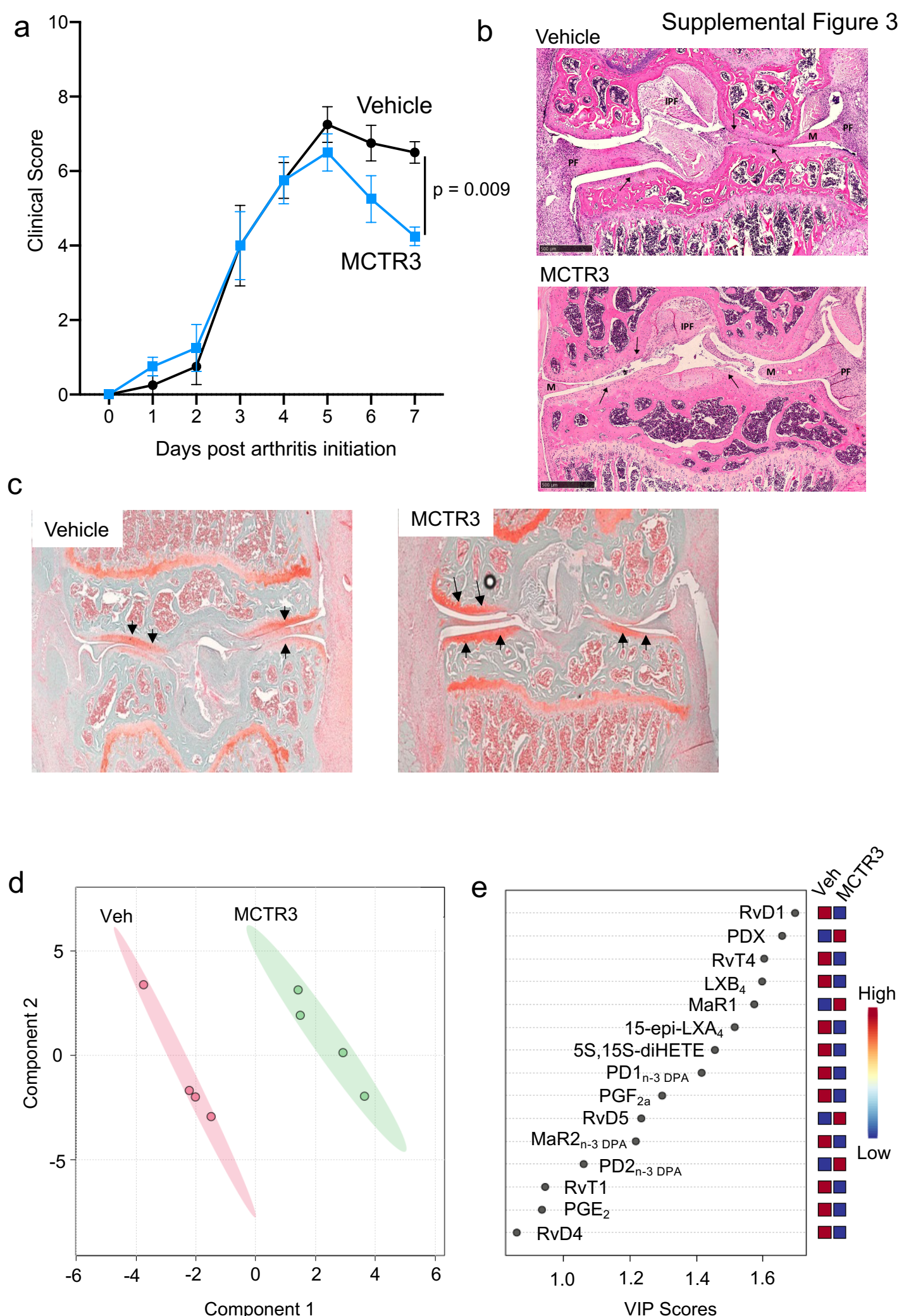

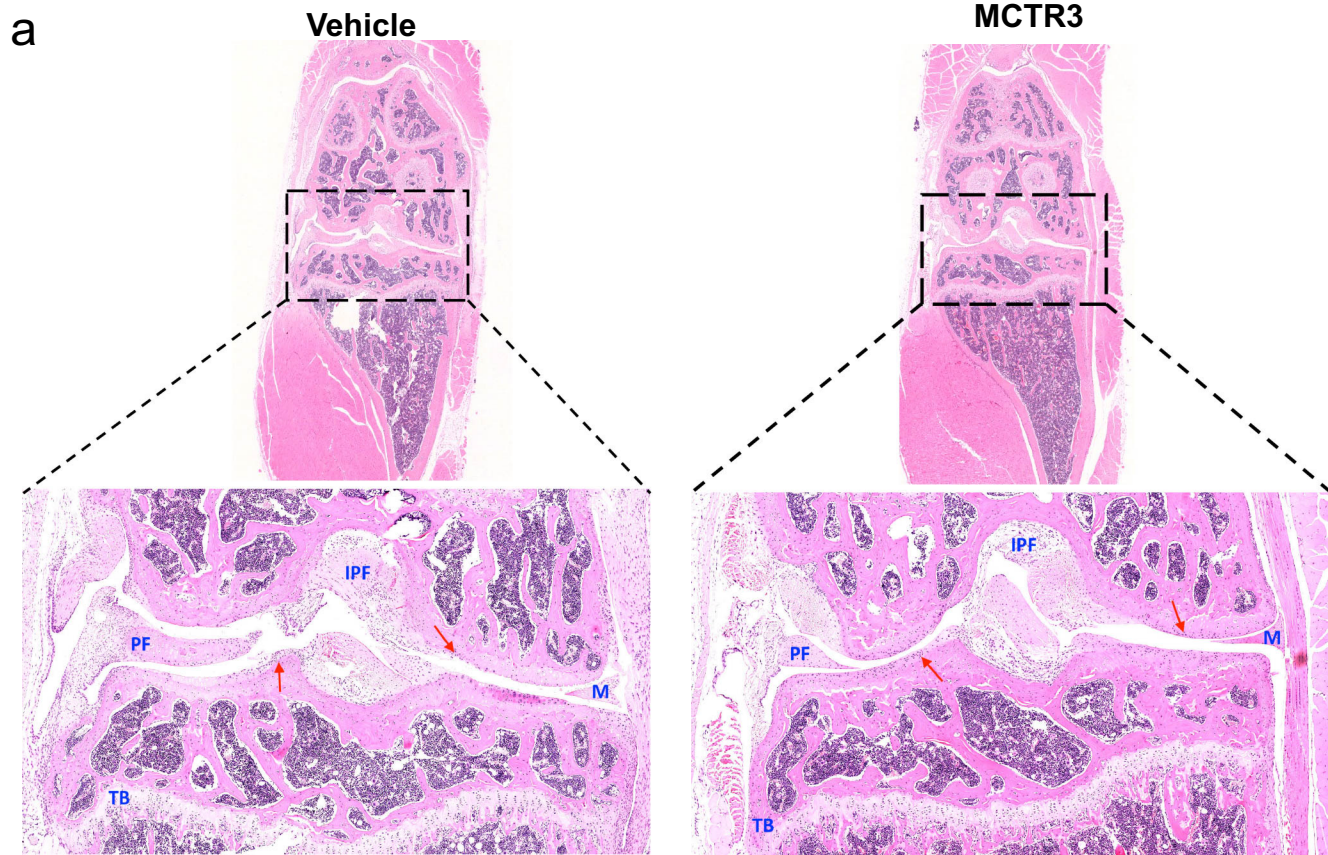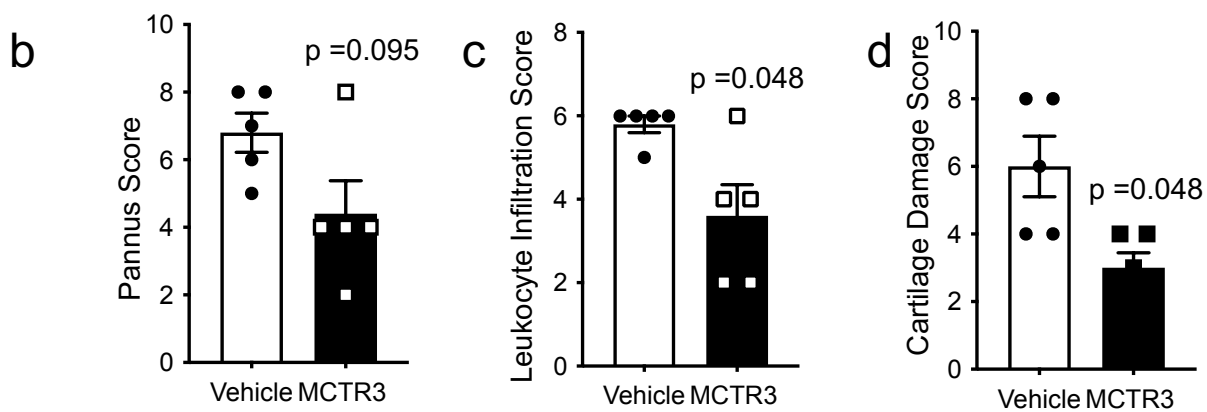

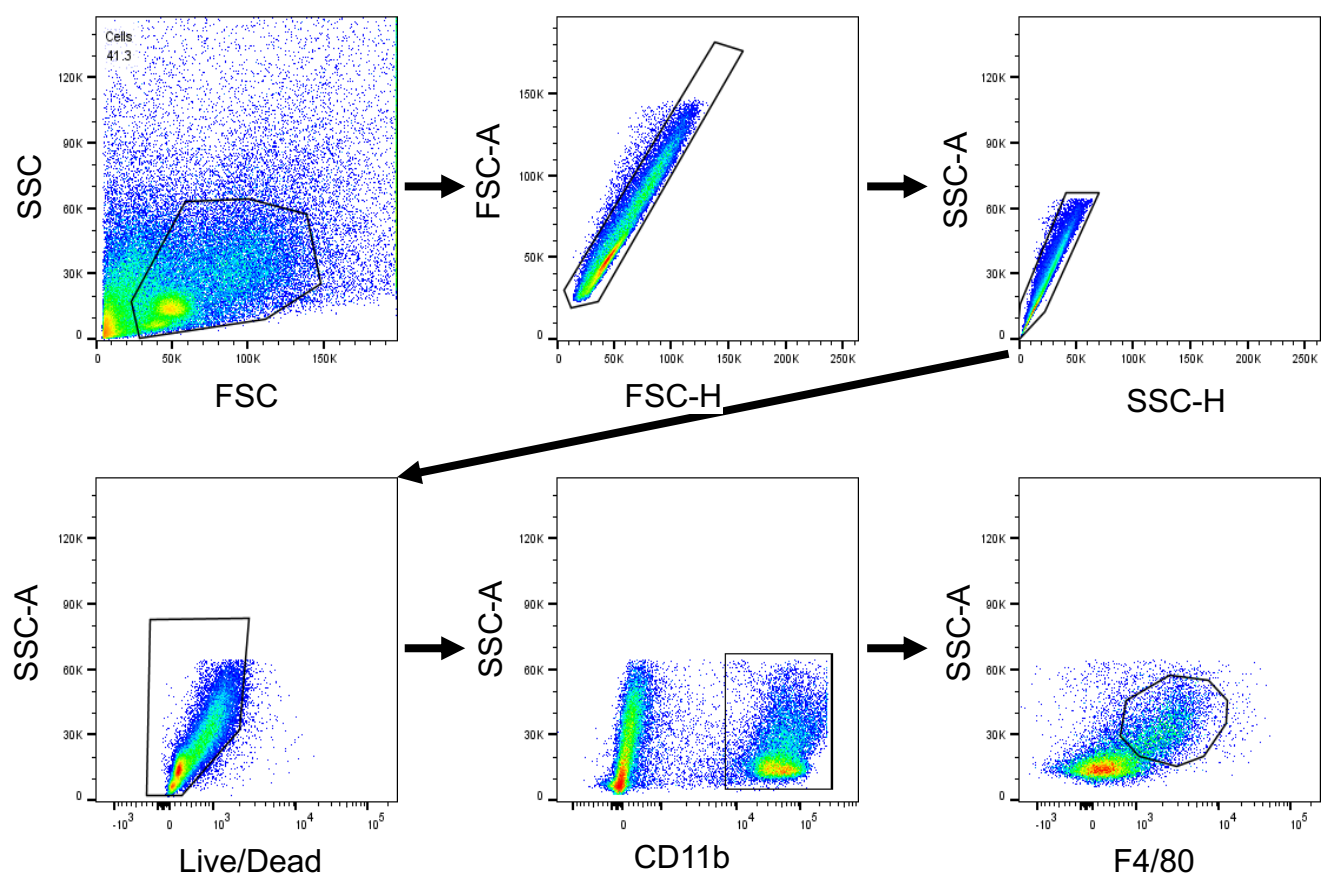

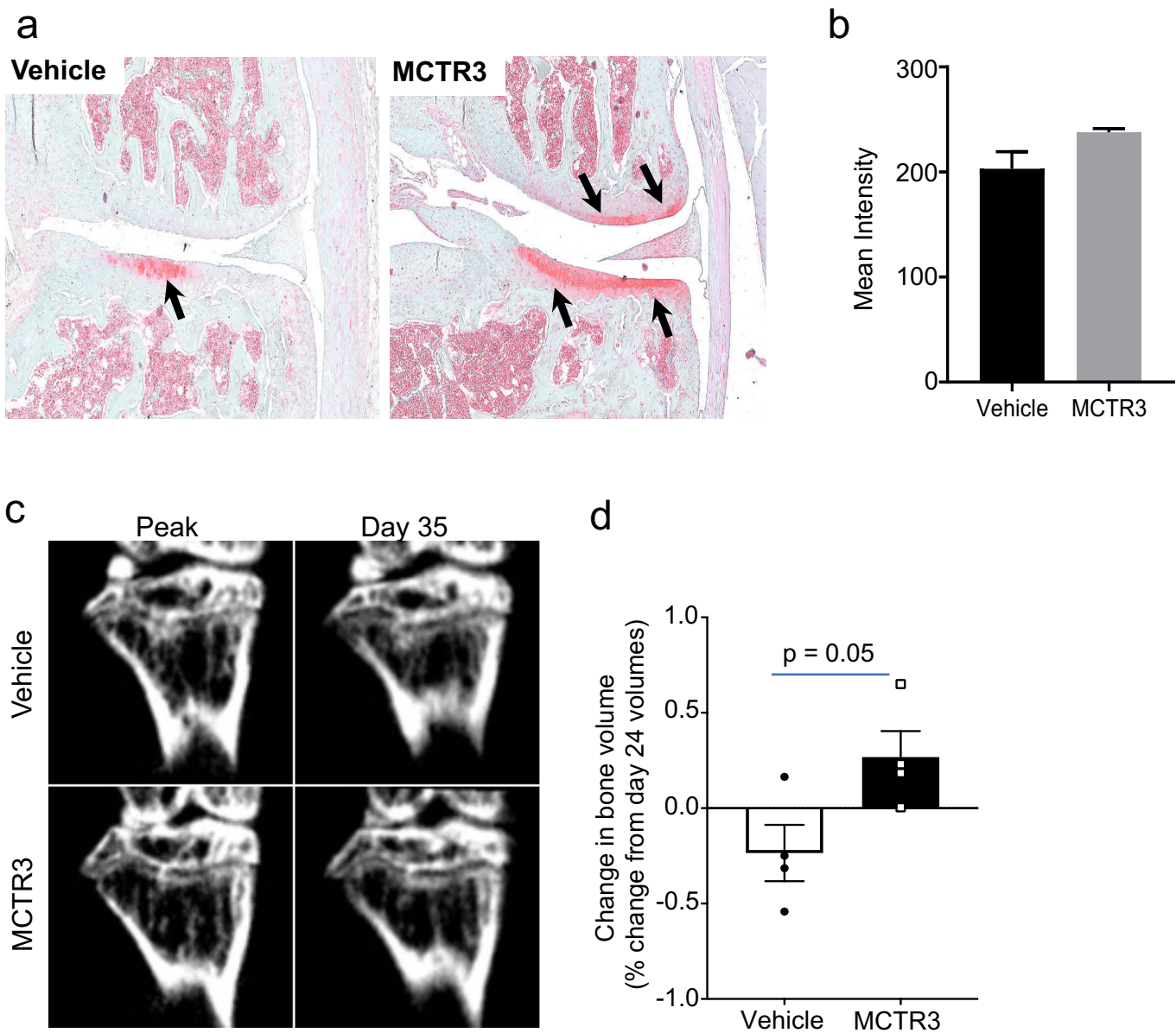

**a**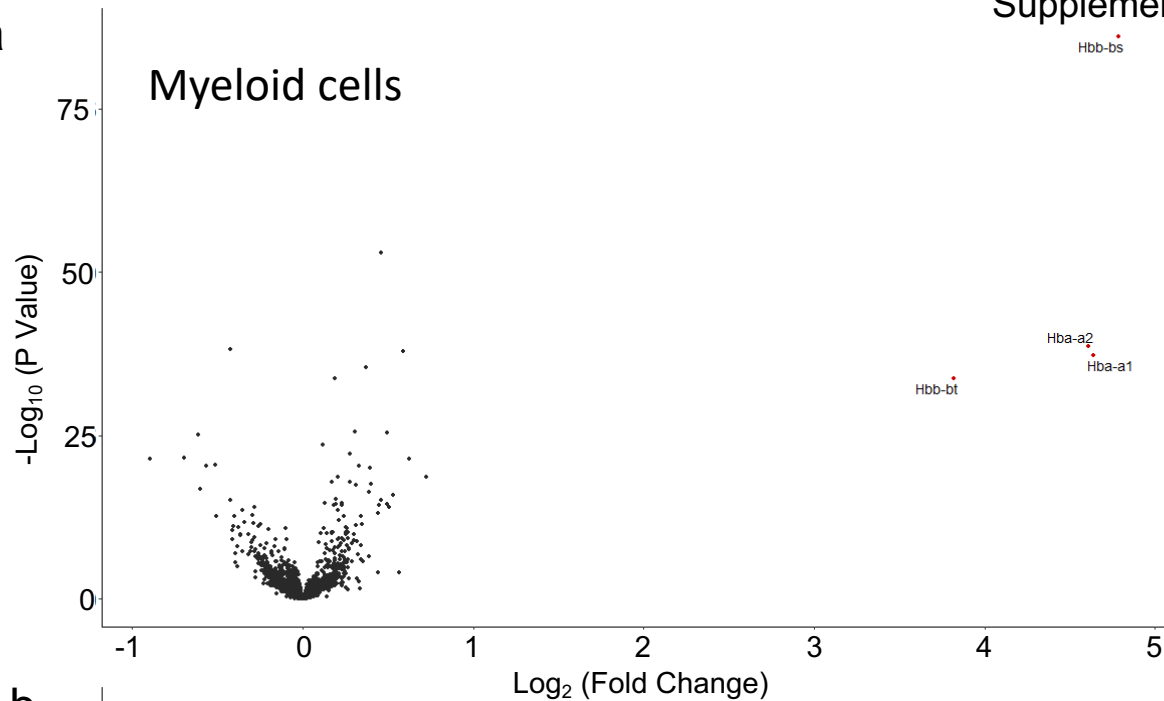**b**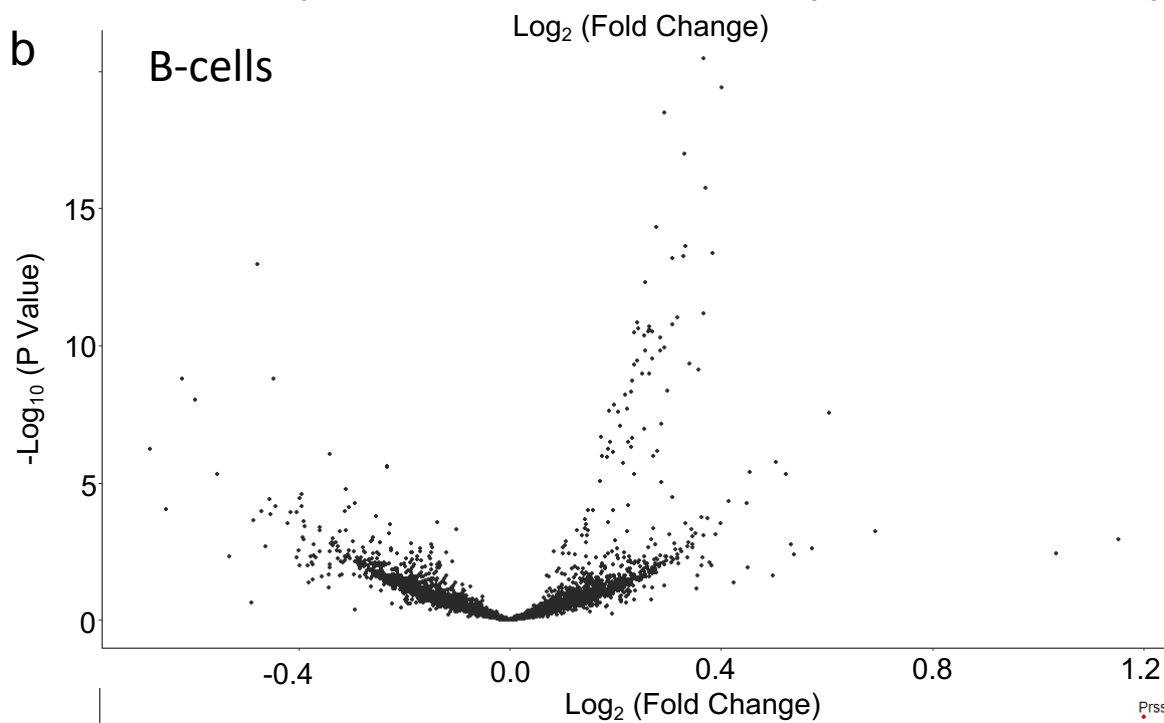**c**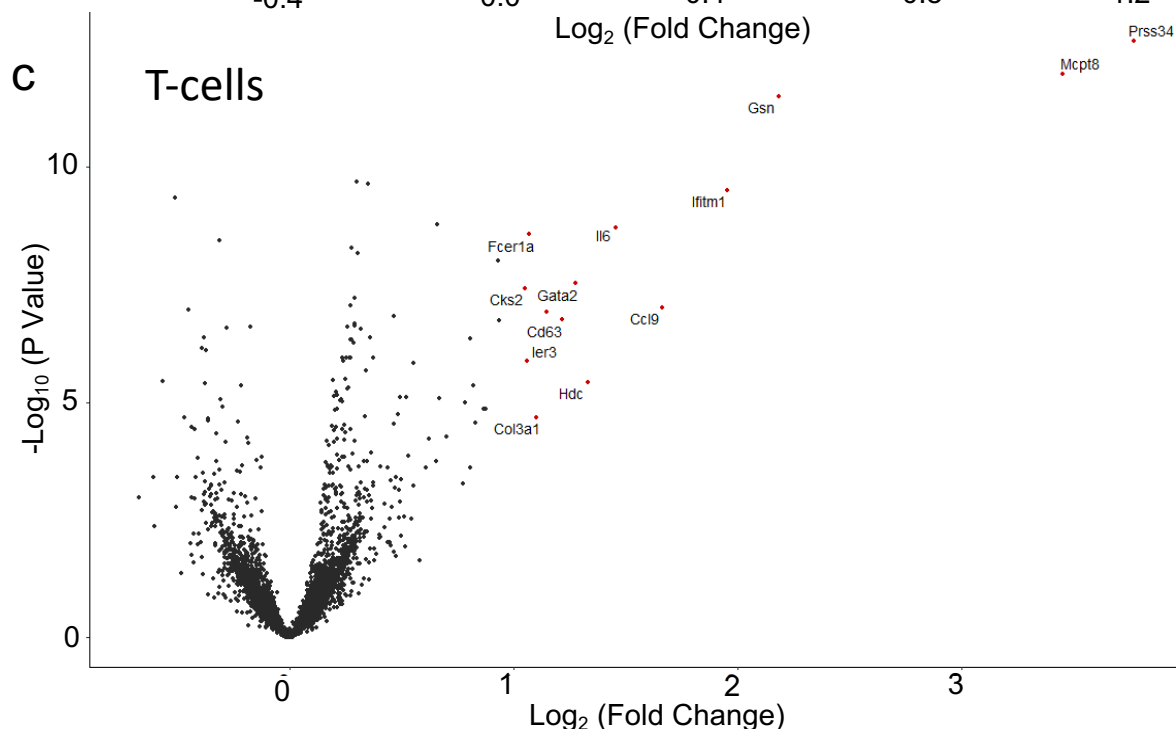

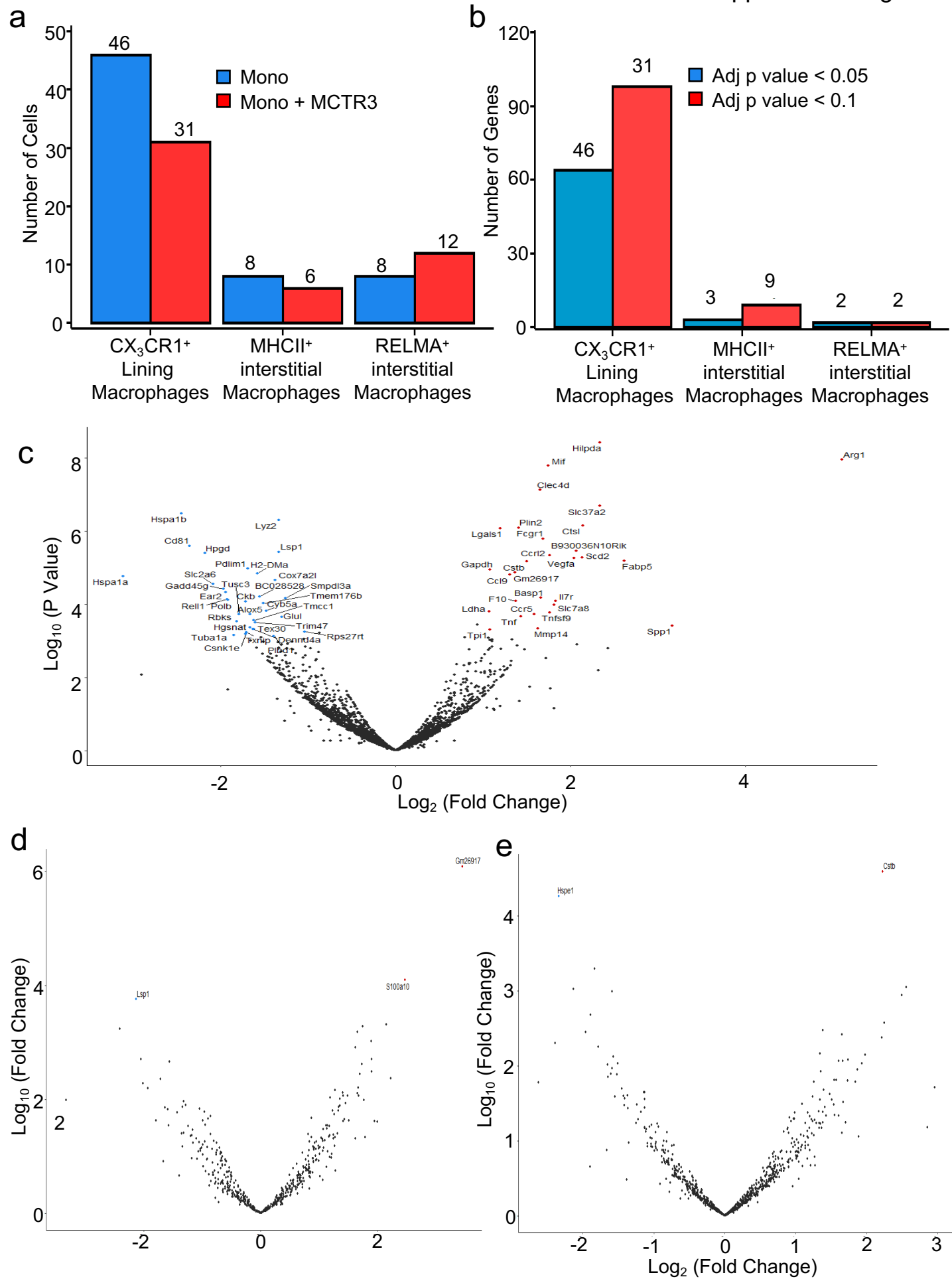

Supplement: Supplementary file 1 [file mmc1.pdf]
