# Supplementary material for: MCTR3 reprograms arthritic monocytes to upregulate Arginase-1 and exert pro-resolving and tissue-protective functions in experimental arthritis
Source: eBioMedicine. 2022 Apr 14;79:103974. doi: 10.1016/j.ebiom.2022.103974 (PMC9038546; doi:10.1016/j.ebiom.2022.103974)
Supplement: Supplementary file 2 [file mmc2.docx]

**Supplementary Legends**

**Figure S1: Plasma MCTR3 are reduced in patients with radiographic signs of erosion and are linked with joint disease phatotype.** Plasma was collected from a patient cohort of DMARD naive patients and concentrations for MCTR1, MCTR2 and MCTR3 were evaluated in relation to (a) joint disease phatotype (b) radiographic evidence of joint erosion (c) responsiveness to DMARD treatment, Non-resp (Non responders) Resp (responders). Results for a are n = 28 patients for Fibroid-pauci and Lymphoid phenotypes and 33 for Myeloid phenotype; for b n = 67 for patients without signs of erosion (no-erosion group) and n = 15 for patients with signs of erosion (erosion group); and for c n = 32 for DMARD non -responders and 67 for DMARD non-responders. (Statistical differences for a were evaluated using Kruskall Wallis test and Dunn’s multiple comparison post hoc test. For b, c statistical difference were evaluated using Mann Whitney U Test).

**Figure S2: Validation of MCTR3**

MCTR3 was obtained via total organic synthesis and the physical properties of the synthetic material (a) UV chromophore, (b) Retention Time in Liquid chromatography and (c) MS/MS fragmentation profile was determined and compared to published values.

**Figure S3: MCTR3 limits joint inflammation.**

Mice were administered K/BxN serum on days 0 and 2 and then treated with MCTR3 (100 ng/mouse) or vehicle (DPBS + 0.1 % EtOH) on days 3 and 5. Disease course was evaluated by assessing (a) clinical scores. (b-c) On day 7 joints were collected and disease severity was evaluated using (b) H&E Staining, arrows denote leukocyte infiltration and (c) glycosaminoglycan content was assessed using Safranin-O staining, arrows denote Safranin-O staining in cartilage. Results for (a) are presented as mean ± SEM. (Statistical differences for (a) were evaluated using Two-Way ANOVA). n = 4 mice per group. IFP = intrapatellar fat, M = meniscus, PF = Pannus formation. (d-e) Lipid mediator profiles were determined using LC-MS/MS-based lipid mediator profiling and evaluated using PLS-DA. (d) scores plot with highlighted regions denoting the clusters representing each group and (e) VIP scores for top 15 mediators. Each dot in the score plot represents a separate mouse.

**Figure S4: MCTR3 decreased leukocyte infiltration and cartilage damage in arthritic mice. Related to Figure 1.** C57BL/6 mice were administered 100 µL K/BxN serum *i.p.* on day 0, 2 and 8. Mice were treated with 1 µg/mouse MCTR3 or vehicle *i.v.* (DPBS + 0.1 % EtOH) on day 10, 12 and 14. Hind paws were collected on day 25, fixed and stained using H&E stain. (a) representative images (b) Pannus size (c) leukocyte infiltration and (d) cartilage damage were evaluated. (Statistical differences were calculated using Mann Whitney U test). Results are mean ± SEM. n = 5 mice per group. IFP = intrapatellar fat, M = meniscus, TB = Tibia, PF = Pannus formation, Red arrows denote leukocyte infiltration.

**Figure S5: Gating strategy employed in the identification of monocyte-derived macrophages.**

**Figure S6: MCTR3 promotes bone and cartilage repair in arthritic mice.**

G6PI peptide (10 µg/mouse) in CFA was administered to DBA/1 mice (100 µL/mouse) and mice were treated with either vehicle (DPBS + 0.1 % EtOH) or 1 µg/mouse MCTR3 *i.v.* on day 24, 26 and 28. On day 36, hind paws were harvested for histological analysis. (a) Representative images from knee joints from a vehicle and MCTR3 treated mouse stained with Safranin-O and (b) quantitative analysis of Safranin O staining. Results are mean ± SEM n = 5 mice per group. (c-(d) microCT analysis was performed *in vivo* on day 24 and 35, to evaluate bone erosion in the arthritic knees. (c) Representative images from the microCT scans from the knee from arthritic mice treated with either vehicle or MCTR3 exhibiting a 2-D coronal cross-sectional image of the proximal tibia, where the ROI was selected in the trabecular epiphysis and (d) quantitative analysis of bone volume. Results are mean ± SEM and expressed as percent change from values obtained on day 24 for each mouse. n = 4 mice per group. (Statistical differences were evaluated using a Mann-Whitney U test).

**Figure S7: Differential gene expression of distinct cell clusters identified in the sc-RNA seq.** K/BxN serum (100 µL, *i.p*.) was administered to mice on days 0, 2 and 9 to induce and prolong inflammatory arthritis. On day 12, mice were treated *i.v.* with 2 x 10^6^ monocytes isolated from arthritic mice that were previously incubated with either vehicle (PBS + 0.1 % EtOH) or 1 nM MCTR3 for 90 min. Cells were isolated from paw joints on day 22, sorted for CD45^+^ cells and single cell RNA sequencing was performed. Volcano plot highlighted differentially regulated genes in the (a) Myeloid cells, (b) B-cells and (c) T-cells population. Results are from n = 4 mice per group

**Figure S8: Differential gene expression in distinct joint macrophages following treatment with MCTR3 reprogrammed monocytes.** K/BxN serum (100 µL, *i.p*.) was administered to mice on days 0, 2 and 9 to induce and prolong inflammatory arthritis. On day 12, mice were treated *i.v.* with 2 x 10^6^ monocytes isolated from arthritic mice that were previously incubated with either vehicle (DPBS + 0.1 % EtOH) or 1 nM MCTR3 for 90 min. Cells were isolated from paw joints on day 22, sorted for CD45^+^ cells and single cell RNA sequencing was performed. (a) the number of cells identified for each cell subsets in mice receiving monocytes (Mono) or MCTR3 reprogrammed monocytes (Mono+MCTR3). (b) the number of genes found to be differentially expressed in the different cell subsets and (c-e) volcano plots for (c) CX_3_CR1^+^ Lining macrophages (d) MHCII+ interstitial macrophages and (e) RELMA+ interstitial macrophages.

**Supplementary Tables**

**Supplemental Table 1: Genes employed for the characterization of distinct cell subsets in sc-RNA seq analysis**

**Supplemental Table 2: Cell counts for the distinct cell subsets identified in the sc-RNA seq.**

**Supplemental Table 3: Cell counts for the distinct cell clusters**

**Supplemental Table 4: Lipid mediator profiles from arthritic joints of MCTR3 treated mice**

K/BxN serum (100 μL, i.p.) was administered to C57BL/6 mice on day 0 and 2 and then treated with MCTR3 (100 ng/mouse) or vehicle (DPBS + 0.1 % EtOH) on days 3 and 5. On day 7 joints were collected lipid mediators were identified and quantified using LC-MS/MS. Results are mean ± SEM from 5 mice per group. - = below limits of quantitation**.**

**Supplemental Table 5: Paw lipid mediator profiles after the administration of arthritic**

**monocytes or MCTR3-reprogrammed monocytes.**

K/BxN serum (100 μL, i.p.) was administered to C57BL/6 mice on day 0, 2 and 9 to induce and prolong inflammatory arthritis and, on day 12, mice were treated i.v. with 2 x 10^6^ monocytes isolated from arthritic mice and incubated with either vehicle (DPBS + 0.1 % EtOH) or 1 nM MCTR3 for 90 min at 37°C. Paws were harvested on day 22 and lipid mediators were identified and quantified using LC-MS/MS. Results are mean ± SEM from 5 mice per group. - = below limits of quantitation**.**

**Supplemental Table 6: Transcript expression of different cell subsets from joint leukocytes from mice treated with monocytes or MCTR3-reprogrammed monocytes.**

**Supplemental Table 7: KEGG Pathway, REACTOME gene sets and GO Biological Processes from mononuclear phagocytes incubated with Vehicle or MCTR3.**
